# *cis*–*trans* Isomerization of silybins A and B

**DOI:** 10.3762/bjoc.10.105

**Published:** 2014-05-08

**Authors:** Michaela Novotná, Radek Gažák, David Biedermann, Florent Di Meo, Petr Marhol, Marek Kuzma, Lucie Bednárová, Kateřina Fuksová, Patrick Trouillas, Vladimír Křen

**Affiliations:** 1 Institute of Microbiology, v.v.i. AS CR, Vídeňská 1083, Prague 4, CZ-14220, Czech Republic; 2 Department of Biochemistry, Faculty of Science, Charles University in Prague, Hlavova 8, CZ-12840 Prague 2, Czech Republic; 3 Inserm UMR-S850, Faculté de Pharmacie, Université de Limoges, 2 Rue du Docteur Marcland, F-87025 Limoges, France; 4 Present address: Department of Physics, Chemistry and Biology (IFM), Linköping University, SE-58183, Linköping, Sweden; 5 Institute of Organic Chemistry and Biochemistry, v.v.i. AS CR, Flemingovo náměstí 2, Prague 6, CZ-16610, Czech Republic; 6 Department of Physical Chemistry, University of Olomouc, tř. 17. listopadu 12, CZ-77146 Olomouc, Czech Republic; 7 Laboratoire de Chimie des Matériaux Nouveaux, Université de Mons, Place du Parc 20, B-7000 Mons, Belgium

**Keywords:** 2,3-*cis-*silybin, 10,11-*cis*-silybin, isomerization, silibinin, silybin, silymarin

## Abstract

Methods were developed and optimized for the preparation of the 2,3-*cis*- and the 10,11-*cis*-isomers of silybin by the Lewis acid catalyzed (BF_3_∙OEt_2_) isomerization of silybins A (**1a**) and B (**1b**) (*trans*-isomers). The absolute configuration of all optically pure compounds was determined by using NMR and comparing their electronic circular dichroism data with model compounds of known absolute configurations. Mechanisms for *cis*–*trans*-isomerization of silybin are proposed and supported by quantum mechanical calculations.

## Introduction

**Figure 1:**
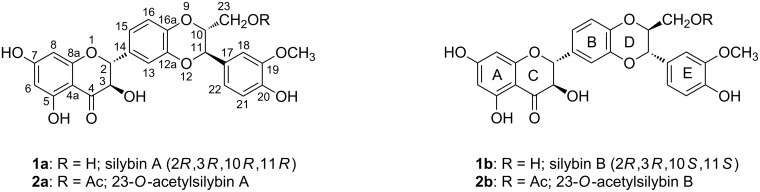
Selected naturally occurring *trans*-silybins and their acetates.

**Figure 2:**
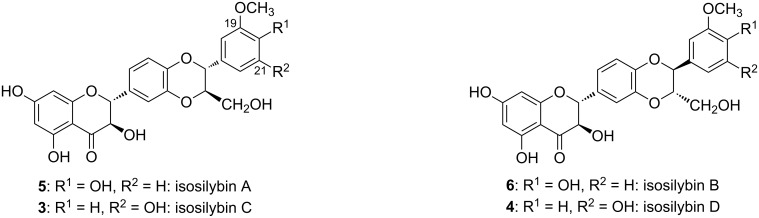
Isosilybins occurring as minor components of silymarin.

The flavonolignan silybin (alternative name silibinin), occurring in the fruits of *Silybum marianum* (milk thistle), consists of two stereoisomers – silybin A (**1a**) and B (**1b**) – in a ca. 1:1 ratio (Figure 1). Their absolute configuration is known [1–2] and their separation was accomplished recently [3–5]. Both silybin isomers as well as other flavonolignans from silymarin (crude defatted extract from the fruits of *S. marianum*) are products of a phenolic oxidative coupling of the flavonoid taxifolin and the lignan coniferyl alcohol. The mechanism of this coupling reaction was described [6] and implied a 10,11-*trans* relative configuration, as in all the major components of silymarin. Little is known about the structures of the minor components of silymarin. It was speculated that some of them were 10,11-*cis*-analogues of the major silymarin constituents [7], but these minor components were never isolated in sufficient amount and purity to enable such unwarranted hypotheses to be verified [8–9]. Another study focusing on the minor components of silymarin identified two new compounds isosilybin C (**3**) and D (**4**), however these were shown to be regioisomers of isosilybins A (**5**) and B (**6**) [10] (Figure 2).

Other authors reported 2,3-*cis*-isomers of silybin [11–12], the relative configuration of which were corroborated by ^1^H NMR coupling constants, i.e., *J_2_*_,_*_3_* of ca. 11 Hz in the *trans*-isomers and 2–3 Hz in the *cis*-isomers. Nevertheless, their absolute configurations remained unknown. The origin of the *cis*-isomers either as biosynthetic side products or as artifacts formed during their isolation is also unknown.

The isolation of naturally occurring *cis*-isomers in the pure form and their complete structure identification would be the only unambiguous way how to prove identity of natural and synthetic *cis*-isomers. Unfortunately, the conventional identification by LC–MS or UV–vis scanning is inadequate as the major and minor compounds exhibit the same MS and UV profiles.

The content of silybin *cis*-isomers in silymarin is presumably very low. The composition of silymarin strongly depends upon its source, which is influenced by the variety of *S. marianum* and by the cultivation, harvest and processing conditions. Variations of the minority content (not only silybin *cis*-isomers) is even more pronounced. Therefore, their identification in a particular silymarin preparation would have only limited information value.

Silybin is an important pharmaceutical commodity and a complete understanding of its composition is needed in order to understand its pharmaceutical properties. Therefore, the detailed structural knowledge and availability of (potential) minor impurities is a fundamental requisite, e.g., for master file assembly.

The aim of this work was to prepare stereochemically pure 2,3-*cis*- and 10,11-*cis*-isomers of silybin A (**1a**) and B (**1b**) by a Lewis acid catalyzed isomerization to determine their absolute configuration and to propose a mechanism for the *cis*–*trans*-isomerization processes.

## Results and Discussion

### Chemistry

**Figure 3:**
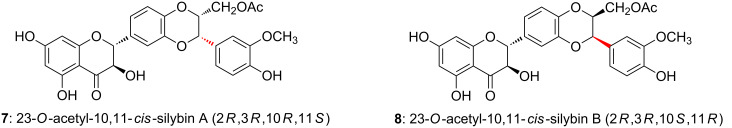
Structures of *cis*-derivatives obtained by the isomerization of **1** using BF_3_·OEt_2_ in EtOAc.

In our previous work on the enzymatic kinetic resolution of silybin [4–5], BF_3_∙OEt_2_ in EtOAc was found to catalyze the transesterification of silybin to yield not only 23-*O*-acetylsilybin (**2**, ca. 90%), but two novel compounds with UV spectra similar to that of silybin. Enzymatic alcoholysis (*n*-butanol) of this mixture by Novozym 435 led, after a prolonged reaction time, to the removal of the acetyl groups from *trans*-isomers **2a** and **2b** to give **1a** and **1b** (Figure 1), while the two minor isomers remained acetylated. Separation of the resulting mixture by silica gel column chromatography yielded an inseparable mixture of the two new compounds. In HPLC they were assigned to two separate peaks with the same molecular mass (*m*/*z* 524). ^1^H NMR spectra indicated that the two compounds differ only slightly from the silybin derivatives **2a** and **2b**, respectively. The unknown compounds exhibit a *J*_10,11_ of ca. 3 Hz, whereas natural silybin (**1**) has a *J*_10,11_ of ca. 8 Hz. Based on this evidence, the unknown compounds from the acylation reaction were proposed to be a diastereoisomeric mixture of 23-*O*-acetyl-10,11-*cis*-silybin A (**7**) and B (**8**) (Figure 3) in a ca. 1:1 ratio.

As the yields of the two 10,11-*cis*-silybin isomers were low (ca. 10%), we optimized the reaction conditions for better yields. In the initial screening, aimed at finding the most suitable solvent and Lewis acid (Table 1), we used natural silybin, i.e., ca. 1:1 mixture of **1a** and **1b**.

**Table 1:** Screening of suitable Lewis and protic acids for silybin isomerization.

Lewis acid	conversion (%)^a^	note

BF_3_·OEt_2_	15–20	
SnCl_4_	12–15	
TiCl_4_	–	strong complexation, quantitative oxidation to 2,3-dehydrosilybin
FeCl_3_	no reaction	strong complexation
ZnCl_2_	no reaction	
BBr_3_	–	decomposition
toluene-4-sulfonic acid	no reaction	

^a^Reaction conditions: silybin, Lewis or protic acid (>10 equiv), DMF, 50 °C, 1–3 h.

The choice of solvent was limited by the low solubility of silybin in most organic solvents or by their incompatibility with the Lewis acid BF_3_·OEt_2_. The following solvents were tested: EtOAc, DMF, CH_3_CN, DMSO, CHCl_3_ at 0, 25, and 50 °C. In CH_3_CN and DMSO silybin decomposed, and in CHCl_3_ the reaction failed. In EtOAc, the reaction was accompanied by C-23 *O*-acetylation, which complicated the reaction mixture analysis. DMF at 50 °C was found to be the most suitable solvent for the isomerization and was used for further Lewis acid screening. Eventually, BF_3_·OEt_2_ proved to be the most suitable Lewis acid for silybin isomerization (Table 1). Other Lewis acids either gave lower isomerization yields (SnCl_4_), did not work at all, or even caused decomposition. Toluene-4-sulfonic acid, as a representative protic acid, gave no reaction under the same conditions.

**Scheme 1:**
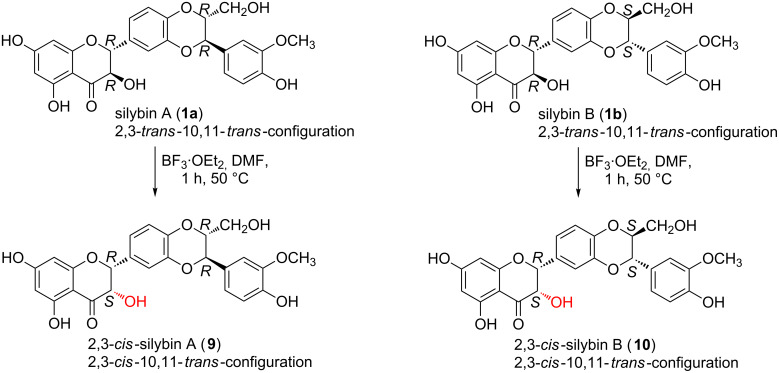
Silybin A and silybin B isomerizations into their 2,3-*cis*-isomers (DMF).

**Scheme 2:**
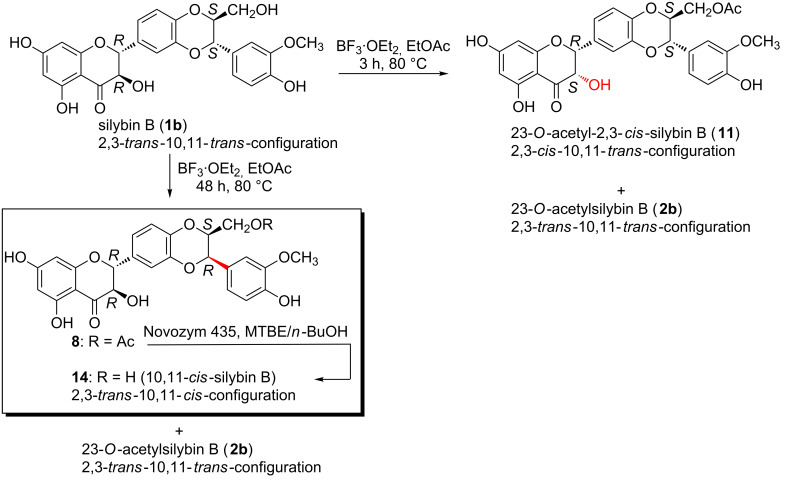
Silybin B isomerization in EtOAc.

**Scheme 3:**
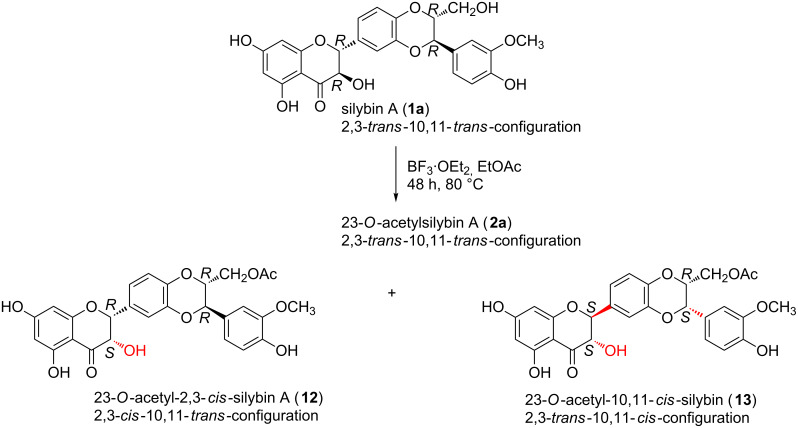
Isomerization of silybin A in EtOAc.

In this work, the preparative reactions with the two solvents DMF and EtOAc and with stereochemically pure silybin A (**1a**) and B (**1b**) (Figure 1) exhibited a strong dependence on the solvent. In DMF (BF_3_·OEt_2_, 50 °C), the reaction led to a single type of C-2, C-3-isomerization yielding the corresponding 2,3-*cis*-10,11-*trans*-silybin A (**9**) and 2,3-*cis*-10,11-*trans*-silybin B (**10**) (Scheme 1), respectively. No C-10, C-11-isomerization was observed in this case. In EtOAc, various *cis*-isomers were obtained, depending on the reaction time and also on the type of starting material (**1a** or **1b**). A detailed analysis of silybin B (**1b**) isomerization in EtOAc showed the formation of the 23-*O*-acetyl-2,3-*cis*-10,11-*trans*-isomer **11** within the first 3 hours, then it slowly disappeared, and finally 23-*O*-acetyl-2,3-*trans*-10,11-*cis*-silybin B (**12**) was formed (Scheme 2). In EtOAc (48 h, 80 °C), **1a** isomerized into the 23-*O*-acetyl-2,3-*cis*-10,11-*trans*-isomer **12** and a minor 23-*O*-acetyl-2,3-*trans*-10,11-*cis*-isomer **13** (Scheme 3). Surprisingly, the absolute configuration at C-2, C-3 of compound **13** was the opposite of **1a** (2*S*,3*S* vs 2*R*,3*R*), which was confirmed by a comparison of their electronic circular dichroism (ECD) data. Formation of the 10,11-*cis*-isomer with the 2*R*,3*R* configuration was not observed during/after the isomerization of **1a** in EtOAc. All the reactions in EtOAc were accompanied by C-23 *O*-acetylation.

### Separation and purification of 2,3-*cis*-silybins

**Scheme 4:**
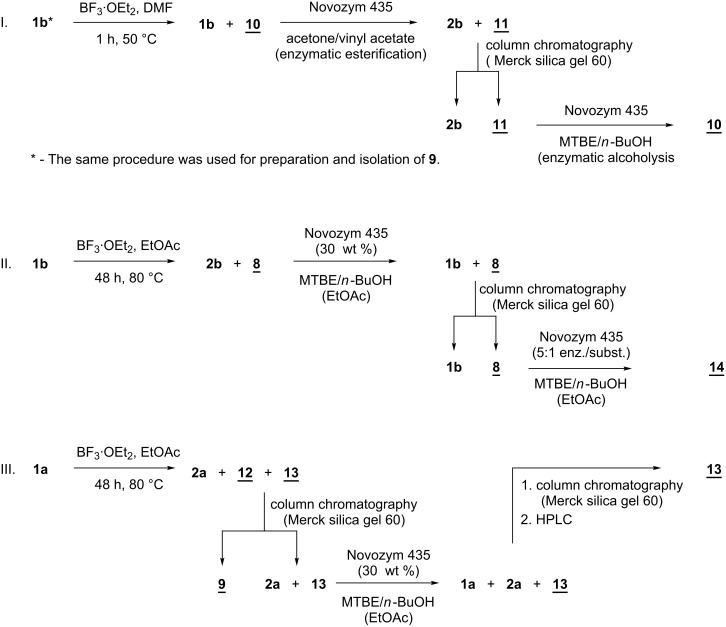
Schematic flowchart of the procedures for the preparation and the isolation of *cis*-silybin isomers (*cis*-isomers are underlined).

The separation of 2,3-*cis*-silybin **7** from unreacted 2,3-*trans*-silybin **1a** (or **10** from **1b**) on silica gel was not feasible, analogous to the preparative separation of silybin stereoisomers **1a** and **1b** (Figure 1), which was a challenge for several decades. It was accomplished as the separation of the respective silybin glycosides for the first time [13–14], later by HPLC [3], and at the preparatory scale by lipase-catalyzed discrimination [4–5]. However, chromatographic separation of 2,3-*cis*- and 2,3-*trans*-silybins is feasible after their C-23 *O*-acetylation, which can be accomplished with Novozym 435 in an acetone/vinyl acetate mixture, giving a nearly quantitative yield of the respective acetates (without the risk of further isomerization) (Scheme 4, part I). As the 2,3-*trans*-isomer was more abundant than the 2,3-*cis*-isomer its excess was removed before enzymatic acetylation by crystallizing the crude mixture from MeOH/H_2_O 9:1, which increased the ratio of the *cis*/*trans*-isomer in the mixture from 1:4 to 1:2.

### Deacetylation of *cis*-23-*O*-acetylsilybins

All pure *cis*-silybins were obtained as their C-23 acetates. The deacetylation proved to be rather difficult, as both acidic and basic hydrolysis as well as mild transesterification (NaOMe, MeOH) failed and/or resulted in decomposition or reversed isomerization to the original *trans*-isomers. We eventually had to use enzymatic alcoholysis with a large excess of Novozym 435 and a longer reaction time (5:1, wt. enzyme/substrate, 3–5 d, 45 °C) compared to the alcoholysis of the respective acetates of the *trans*-silybins. This method finally yielded pure 2,3-*cis*-silybins A (**9**) and B (**10**) as well as 10,11-*cis*-silybin B (**14**) (Scheme 4, parts II and III).

#### Determination of absolute configuration of *cis*-silybins

The determination of the absolute configuration of the new *cis*-silybins by X-ray analysis is not possible, since all attempts to obtain suitable crystals using various crystallization conditions were unsuccessful. Pure silybins and their congeners are known for their poor crystallization or the formation of small crystals, which are not suitable for X-ray analysis. The absolute configuration of silybins A (**1a**) and B (**1b**) was determined indirectly on the basis of a comparison of their physicochemical data (NMR, ECD, OR). Later, these data were matched to those of their 10,11-regioisomer, isosilybin A (**5**) [11]. The same approach was used to determine the absolute configuration of isosilybin B (**6**). The only analogue that yielded suitable crystals, and hence an unequivocal determination of its absolute configuration, was 7-(4-bromobenzoyl)isosilybin A [15].

In this study the determination of the absolute configuration was based on NMR and ECD spectroscopy. Their relative configurations, i.e., the determination of *trans*/*cis* isomers at C-2, C-3 and C-10, C-11, are set by the ^1^H NMR coupling constants *J*_2,3_ and *J*_10,11_, respectively. The *trans*-isomers exhibit *J*_2,3_ of ca. 11 Hz and *J*_10,11_ of ca. 8 Hz, while the *cis*-configuration is indicated by a value of *J*_2,3_ or *J*_10,11_ of ca. 2–3 Hz [11–12].

**Figure 4:**
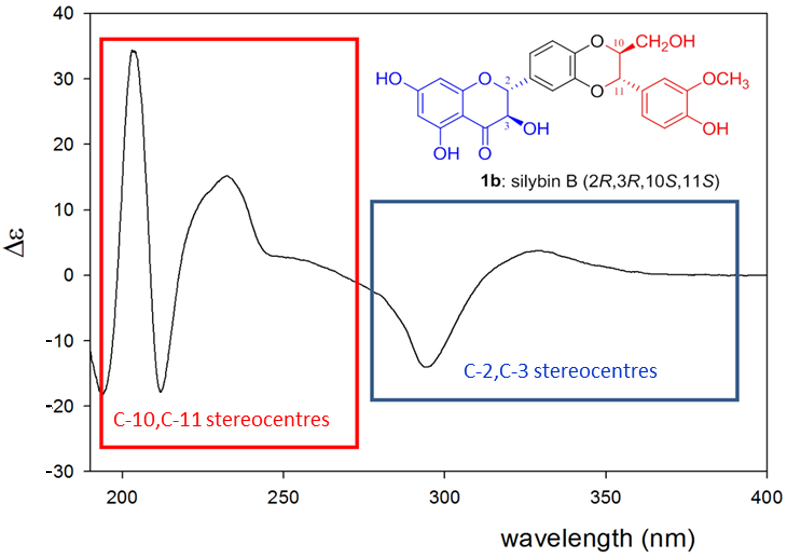
ECD spectrum of silybin B (**1b**) and its separation (in a crude approximation) into two π-conjugated moieties (3-hydroxyflavonone with stereogenic centers C-2, C-3 (in blue) and 1,4-benzodioxane with C-10,C-11) (in red) according to [2].

**Figure 5:**
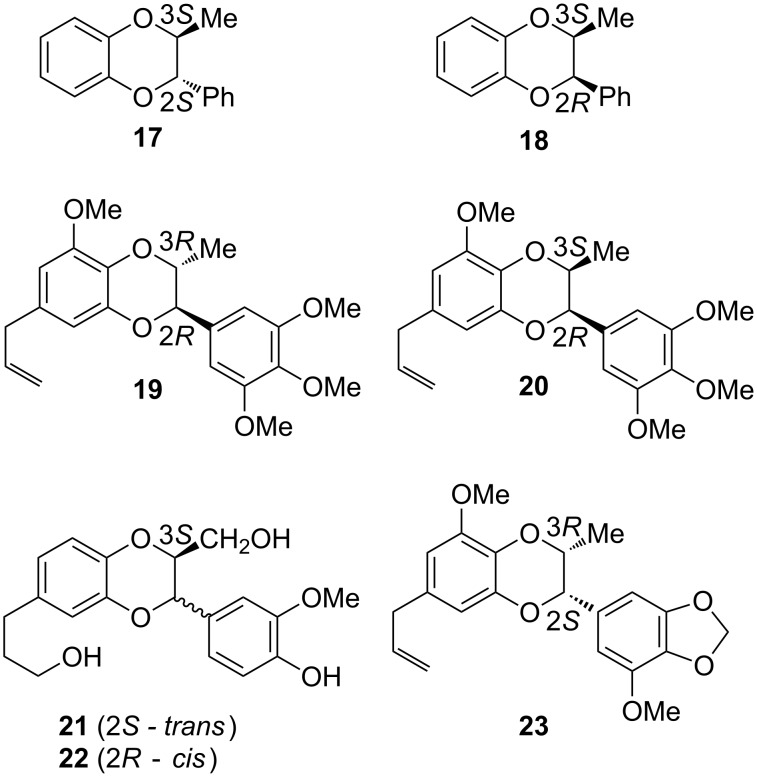
Synthetic and natural (benzodioxane-type) compounds related to silybin stereoisomers with known absolute configurations.

For the assignment of the absolute configuration, experimental ECD spectra of compounds **9**, **10**, **13**, and **14** were compared to those of related compounds with known absolute configurations [2]. The assignment of Cotton effects (CE) to particular regions in this molecule is based on the rough assumption that silybin and its analogues are considered to be composed of two separate π-conjugated subsystems, the 3-hydroxyflavonone moiety (with the stereogenic centers C-2 and C-3), and the 1,4-benzodioxane moiety (C-10 and C-11) (see Figure 4). In this simplification we cannot avoid the adverse effects caused by neglecting the effects of local perturbations on specific stereogenic centers. Thus, the interpretation of our experimental ECD spectra is mainly based on empirical comparison and correlation to those of i) the dihydroflavonols taxifolin (**15**) and epitaxifolin (**16**) isolated from *Thujopsis dolobrata* [16] and ii) various benzodioxanes, e.g.*,* the pair of benzodioxanes **17** and **18** synthesized from (+)-ephedrine; the natural benzodioxanes eusiderin (**19**) and eusiderin C (**20**) [17]; **21** and **22**, isolated from *Juniperus chinensis* [18]; and benzodioxane **23** from *Licaria chrysophylla* [19] (Figure 5), for both the C-2, C-3 and C-10, C-11 pairs, respectively [20].

The ECD of the 3-hydroxyflavonone moiety was discussed with the aim to determine absolute configuration [21]. UV absorption bands in the respective 270–290 nm and 330–320 nm ranges were utilized, which were clearly rationalized in terms of the corresponding electronic transitions [22–23]. A pair of positive–negative CEs at both spectral ranges is characteristic of the (2*R*,3*R*) configuration of the 3-hydroxyflavonone moiety. Nevertheless, it should be kept in mind that there are also other absorption bands at shorter wavelengths giving rise to observable CEs near 230 nm, which are relatively constant in pattern [24–28].

The ECD spectrum of compound **10**: (θ [deg·cm^2^·dmol^−1^], CEs: 339 nm, θ = +24971; 294 nm, θ = −50028; 237 nm θ = +16914; 224 nm, θ = −8082; 213 nm, θ = −44628; 203 nm, θ = +162457; 193 nm, θ = −71457) (Figure S1, Supporting Information File 1) displayed the coupling constant *J*_2,3_ = 2.5 Hz corresponding to a *cis-*configuration and, therefore, compound **10** was assigned to the 2*R*,3*S*-isomer. The ECD spectrum of compound **9**: (θ [deg·cm^2^·dmol^−1^], CEs: 338 nm, θ = +26771; 292 nm, θ = −60650; 233 nm, θ = −58000; 214 nm, θ = −9828; 207 nm, θ = +83000; 197 nm, θ = +77685) (Figure S2, Supporting Information File 1) together with the *J*_2,3_ coupling constant of 2.4 Hz (*cis*-configuration) are similar to (+)-epitaxifolin [16]. Therefore, the absolute configuration of **9** at C-2, C-3 is 2*R*,3*S*. The absolute configuration of compound **14** at C-2, C-3 is analogous to (+)-taxifolin (2*R*,3*R)*, according to its *J*_2,3_ value of 11.4 Hz and its ECD spectrum (**14**: θ [deg·cm^2^·dmol^−1^], CEs: 327 nm, θ = +9568; 293 nm, θ = −46824; 240 nm, Δε = +36917 deg·cm^2^·dmol^−1^; 230 nm, θ = −8890; 211 nm, θ = −63590) (Figure S3, Supporting Information File 1). Accordingly, its absolute configuration at C-2, C-3 should be 2*R*,3*R*. Interestingly, compound **13** seems to be 2,3-*trans*-10,11-*cis*-silybin according to its NMR spectra (*J*_2,3_ = 11.3 Hz, *J*_10,11_ = 2.9 Hz). However, its ECD spectrum (**13**: θ [deg·cm^2^·dmol^−1^], CEs: 328 nm, θ = −12870; 294 nm, θ = +44453; 253 nm, θ = −7535; 242 nm, θ = +7451; 229 nm, θ = −71888; 205 nm, θ = −1116600; 194 nm, θ = +62990) (Figure S4, Supporting Information File 1) corresponds to (−)-taxifolin. According to these results, the absolute configuration at C-2, C-3 is the opposite of the starting compound **1a** (2*R*,3*R*). Therefore, the absolute configuration of **13** is 2*S*,3*S*.

To assign the absolute configuration at C-10 and C-11 of silybin we combined the corresponding coupling constants *J*_10,11_ with the CEs in 200–280 nm spectral range. It was shown that a sign of the CE at ca. 236 nm could be used to determine the stereochemistry at C-3 (i.e. C-10 of silybin) (negative CE corresponds to the *R* configuration) [2,17,20]. Thus, the negative CE around 230 nm for **13**, corroborated by the vicinal ^1^H–^1^H coupling constants (*J*_10,11_ = 2.9 Hz, e.g., *cis*-configuration) (Figure S4, Supporting Information File 1) indicates an absolute configuration of 2*S*,3*S*,10*R*,11*S*. In contrast to the ECD spectrum of silybin B (**1b**) (Figure S3, Supporting Information File 1), the *J*_10,11_ coupling constant (2.8 Hz, *cis*-configuration) of compound **14** and its ECD spectrum (positive/negative CE around 240 nm) implies the absolute configuration at C-10, C-11 to be 10*S*,11*R*, so that the absolute configuration of **14** is 2*R*,3*R*,10*S*,11*R*.

The absolute configuration at C-10, C-11 of the 2,3-*cis*-10,11-*trans*-silybins **9** and **10**, as inferred from the coupling constants (*J*_10,11_ ca 8 Hz, *trans*-configuration) and the ECD spectra (Figure S1 and Figure S2, Supporting Information File 1), is 2*R*,3*S*,10*R*,11*R* (**9**) and 2*R*,3*S*,10*S*,11*S* (**10**). Here we would like to stress that the absolute configuration at C-10, C-11 of **9** and **10** remains the same as in the starting compounds, namely **1a** (10*R*,11*R*) and **1b** (10*S*,11*S*), respectively.

#### Mechanism of silybin isomerization

Based on the absolute configuration of the new *cis*-silybin isomers, we propose mechanisms for the stereospecific isomerization.

**Scheme 5:**
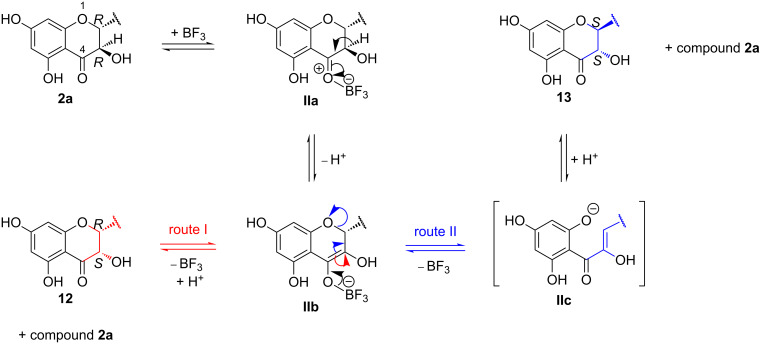
Proposed mechanisms of Lewis acid catalyzed isomerization at the benzopyranone ring of 23-*O*-acetylsilybin A (**2a**) in EtOAc.

The isomerization process is initiated by BF_3_ complexation. Boron trifluoride may complex silybin through a coordinate bond, in which the two electrons originate from the oxygen atoms of silybin. Silybin and BF_3_ are hard bases (η = 4.8 eV in EtOAc) and acids (η = 7.3 eV in EtOAc), respectively (Table 2), favoring such a coordinate bond. The hardness of BF_3_ and silybin A is not significantly modified when the solvent polarity is increased (Table 2). The hardness of silybin B was not calculated, as the stereochemistry is not expected to significantly modify this parameter. The hardness calculation is based on the HOMO (highest occupied molecular orbital) and LUMO (lowest unoccupied molecular orbital) energies, which are the same for silybin A and B. The quantum calculations show that the coordination complex at C-4=O (**IIa** in Scheme 5) is the most stable (Table 3). The corresponding complex **IIa** exhibits a strong O–B bond of 1.55 Å, lower than, e.g., 1.73 Å in the complex at O-1 (Table 4). In the benzopyranone moiety, **IIa** is more stable than the complex at O-1 by ca. 6 kcal·mol^−1^. In the benzodioxane moiety, complexation at O-12 is favored (compared to O-9) by 4 kcal·mol^−1^. The coordination with boron initiates isomerization at C-2, C-3 and C-10, C-11 by charge rearrangements (e.g., proton release and formation of **IIb** in Scheme 5). The mechanisms related to both sites can be considered separately, as both moieties are electronically independent.

**Table 2:** Chemical hardness (η, eV) of BF_3_ and silybin A (**1a**), in three different solvents (obtained at the IEFPCM B3P86/6-31+G(d,p) level of theory).

solvent	η	

	BF_3_	silybin A

benzene	7.4	4.7
EtOAc	7.3	4.8
DMF	7.0	4.8

**Table 3:** Electronic energies (Δ*E*, kcal·mol^−1^) of BF_3_ complexation with silybin A (**1a**) at the different O-atoms.

	EtOAc	DMF

O-1	−6.3	−6.8
C-4=O	−12.4	−12.8
O-9	−6.9	−7.7
O-12	−10.9	−11.3

**Table 4:** Bond distance and atomic charge of interest in BF_3_-silybin A at C-4=O (complex **IIa**) and O-1, in a) EtOAc and b) DMF.

a)	complex at O-1	complex at C-4=O (**IIa**)

d(O–B)	1.734	1.557
d(O1–C2)	1.485	1.441
atomic charge at C3	0.023	0.030

b)	complex at O-1	complex at C-4=O (**IIa**)

d(O–B)	1.718	1.553
d(O1–C2)	1.486	1.442
atomic charge at C3	0.035	0.010

**Figure 6:**
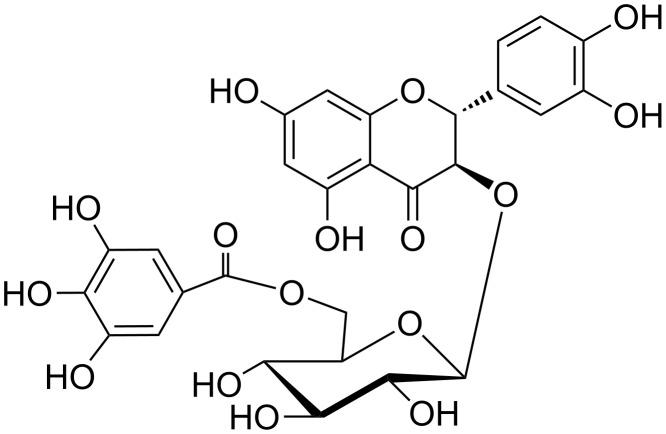
Taxillusin, (2*R*,3*R*)-taxifolin 3-β-D-glucopyranoside 6''-gallate (**24**).

The isomerization of flavanonols at C-2, C-3 is not without precedent, as it was described for taxillusin – the flavanonol glycoside from *Taxillus kaempferi* (Japanese mistletoe) containing the 3-hydroxy-2,3-dihydro-2-phenylchromen-4-one moiety [29]. Taxillusin (**24**, (2*R*,3*R*)-taxifolin 3-β-D-glucopyranoside 6''-gallate, Figure 6) was subjected to both, basic and acidic hydrolysis during its structure elucidation. However, under these conditions four stereoisomers of the original aglycon (2*R*,3*R*)-taxifolin were formed. Base-catalyzed isomerization of another taxifolin glycoside, (2*R*,3*R*)-2,3-dihydroquercetin-3-α-L-rhamnopyranoside (pyridine; aqueous solution), was reported earlier [30–33]. The thermal or enzymatic rearrangement of taxifolin to alphitonin yielded four taxifolin isomers [34]. With flavanonols (3-*O*-glycosides or the respective aglycons), isomerization can be explained by the benzopyranone ring enolization, which can be initiated by either acids or bases. A resonance effect can further cause reversible C-ring opening, thus allowing isomerization at C-2.

The proposed mechanism for the isomerization of silybin at C-2, C-3 was inspired by these previous studies, i.e., involving an enolization stage (route II in Scheme 5) [29–34]. In EtOAc, the isomerization can occur from **1a** or from its acetylated counterpart (**2a**), since the 23-*O*-acetylation proceeds in parallel with the isomerization and is substantially faster than the isomerization reaction.

In principle, the isomerization can be initiated by the formation of coordination complexes at either C-4=O or O-1 (see Scheme 5). Nevertheless, based on the quantum chemical calculations (Table 3 and Table 4) together with the representation of *cis*-isomers in the isomerization mixture, the most stable coordination complex **IIa** at C-4=O is stabilized by a strong O–B bond of 1.55 Å, having an atomic charge at C-3 close to zero (Table 4). This favors a proton release from this position and the formation of the negatively charged species **IIb**. The release of BF_3_ probably occurs from **IIb** by two different routes. The major pathway (route I) proceeds without ring opening and after re-protonation it leads to the major *cis*-isomer **12**. The minor route (route II), accompanied by a ring opening of the benzopyranone, proceeds via intermediate **IIc**, which after ring closure and protonation yielded a mixture of **2a** and **13**. This reversible benzopyranone ring opening/closure process is probably responsible for the inversion of the absolute configuration at C-2, C-3 of **13** compared to **2a** (Scheme 5).

The limited degree of isomerization at C-2 of silybin requiring the ring opening of the benzopyranone is in agreement with a related study of taxifolin isomerization [34]. A deuterium incorporation NMR study supported by quantum chemical calculations showed that the intermediate of taxifolin with an open benzopyranone ring (α-hydroxychalcone related to **IIc** in our study) is very short-lived and the energy barrier for its formation is relatively high [35]. It must be noted that **IIc** contains additional substitution at the *ortho*-dihydroxyphenol moiety, which probably further decreases the possibilities for resonance stabilization compared to unsubstituted α-hydroxychalcone (quinone methide formation is disabled).

Interestingly, the course of **1a** and **1b** isomerization was slightly different in DMF than in EtOAc, since it only yielded the *cis*-isomers **9** and **10**. The isomer with an inverted absolute configuration at C-2, C-3 (the non-acetylated form of **13**) was not observed at all. This is easily explained by the charge distribution of complex **IIa**. In DMF, the atomic charge at C-3 is closer to zero than in EtOAc (Table 4). This clearly indicates that the formation of the open intermediates (**IIc**) is not supported in DMF.

**Scheme 6:**
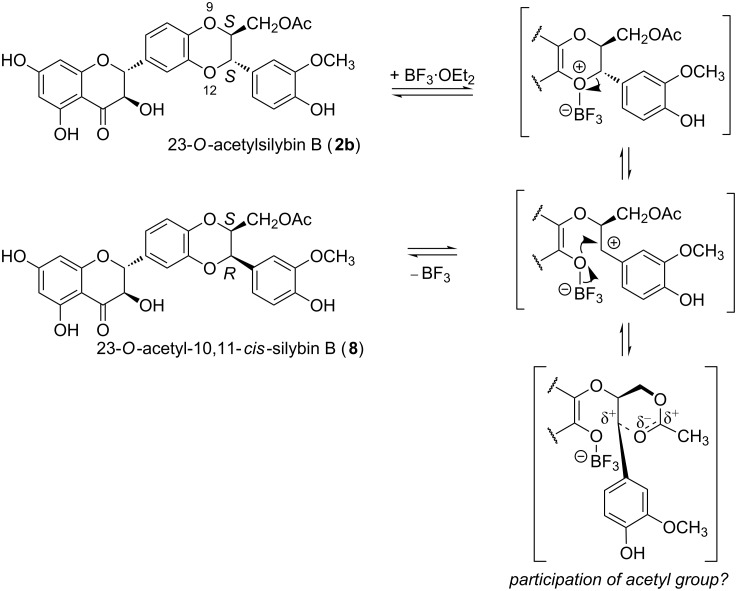
Proposed mechanism of Lewis acid catalyzed isomerization of benzodioxane part of 23-*O*-acetylsilybin B (**2b**) in EtOAc.

**Figure 7:**
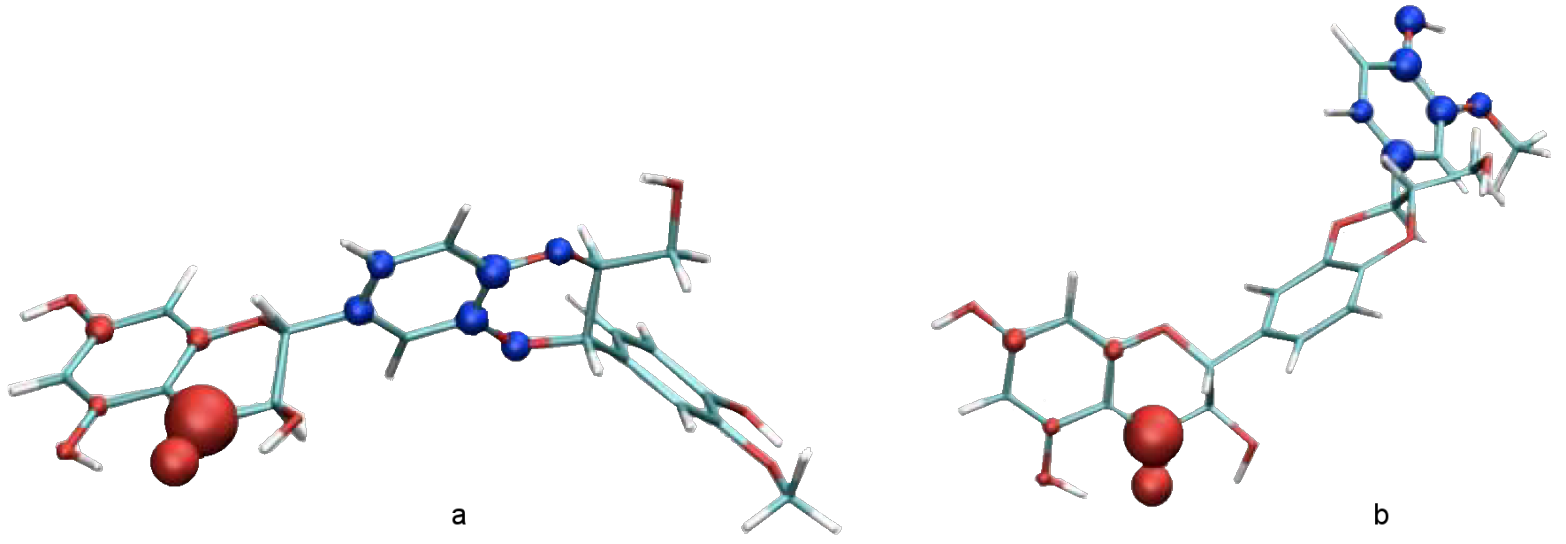
Spatial distribution of nucleophilic *f*−(r) (blue) and electrophilic *f*+(r) (blue) Fukui functions of silybin A (**1a**) in both a) EtOAc and b) DMF.

The isomerization of **1** at the benzodioxane moiety is also initiated by BF_3_ complexation, followed by a multistep mechanism including the ring opening of benzodioxane. As the change in configuration takes place exclusively at C-11, it is probably initiated by the acid-catalyzed dioxane ring opening at O-12 (Scheme 6). This site is highly nucleophilic in EtOAc (Figure 7a), with lone electron pairs, which is in favor of BF_3_ complexation as confirmed by the complexation energies (Table 3). Following complexation, the D-ring opening proceeds (Scheme 6), allowing molecular rearrangements and isomerization after ring closure.

The observation of the isomerization of **1** (or **2**) at C-11 that occurs in EtOAc but not in DMF can be explained either by a solvent effect or by the participation of the acetyl group. However, the second possibility is less probable, since the participation of the acetyl group should lead to the formation of an isomer with a retained configuration at C-11 (two subsequent nucleophilic substitutions with two Walden inversions). The possibility of acetyl group participation was refuted by the reaction of **2** in DMF, so that no isomerization took place at C-11. Moreover, in DMF the *f*(r) Fukui function is totally displaced into the E-ring (Figure 7b), so that the O-12 atom loses its nucleophilic character, which partially rationalizes the solvent effect observed here.

## Conclusion

We report here the first syntheses of hitherto undescribed *cis*-isomers of silybin in optically pure form starting from silybin A (**1a**) and B (**1b**). The determination of their absolute configuration was based on an analysis of NMR coupling constants and a comparison of their ECD spectra with model compounds with well-defined absolute configurations. Moreover, the absolute configuration of these novel 2,3-*cis*- and 10,11-*cis*-isomers of silybin enabled us to propose mechanisms for the *cis*–*trans* isomerization of silybin. Although analogous isomerizations of similar compounds at respective C-2, C-3 centers have been described, we present here parallel isomerizations on both chiral centers under kinetic control. *cis*-Silybins were obtained by the chemoenzymatic separation methods mostly due to the sensitivity of new *cis*-derivatives to the reversed isomerization.

## Experimental

### General experimental procedures

NMR spectra were recorded in DMSO-*d*_6_ (30 °C) by using a Bruker AVANCE 600 NMR spectrometer (600 MHz for ^1^H, 151 MHz for ^13^C) with the residual solvent peak as the internal standard. Mass spectra were measured in an APEX-Ultra FTMS with ESI ionization. The high-resolution ESI–MS spectra were measured by using a GCT Premier benchtop orthogonal acceleration time-of-flight mass spectrometer.

Optical rotations were measured with a Rudolph Autopol polarimeter in acetone at 25 °C, and ECD spectra were recorded in a Jasco-815 spectrometer in MeOH from 200 to 400 nm with a scanning speed 20 nm/min, time response 8 s with a 2 mm quartz cell and a sample concentration of ca. 1 mmol/L.

HPLC analyses were carried out in a Shimadzu Prominence LC analytical system consisting of a Shimadzu LC-20AD binary HPLC pump, Shimadzu SIL-20AC cooling autosampler, Shimadzu CTO-10AS column oven, and Shimadzu SPD-20MA diode array detector. The monolithic column was a Chromolith Performance, RP-18e, 100 × 3 mm equipped with a guard column (5 × 4.6 mm) with an isocratic mobile phase CH_3_CN/MeOH/H_2_O/HCO_2_H 2:37:61:0.1, flow rate 1.5 mL/min at 25 °C, detection at 285 nm; *t*_r_ (silybin A, **1a**) = 3.1 min, *t*_r_ (silybin B, **1b**) = 3.7 min, *t*_r_ (23-*O*-acetylsilybin A) = 12.7 min, *t*_r_ (23-*O*-acetylsilybin B, **2b**) = 14.1 min. For the semipreparatory HPLC, a chromolith SemiPrep RP-18e monolithic column, 100 × 10 mm was used with an isocratic mobile phase CH_3_OH/H_2_O 50:50. The flow rate was set to 5 mL/min at 25 °C, the UV detection to 285 nm; *t*_r_ (**2a**) = 6.6 min, *t*_r_ (**13**) = 7.8 min.

### Materials

Silybin (a mixture of diastereoisomers A (**1a**) and B (**1b**), ca. 1:1) was kindly provided by Dr. L. Cvak (division TAPI, Teva Czech Industries s.r.o., IVAX Pharmaceuticals, CZ). Silybin diastereoisomers were separated by lipase-catalyzed discrimination [5]. *Candida antarctica* lipase B (Novozym 435) was from Novozymes (DK). All other reagents were of analytical grade and used without further purification.

**(2*R*,3*S*,10*R,*11*R*)*-*23-*O*-Acetylsilybin (12):** BF_3_·OEt_2_ (2 mL, 15.926 mmol, 50% solution in OEt_2_) was added to a stirred solution of **1a** (2 g, 4.146 mmol, >99% optical purity) in DMF (15 mL) and further stirred for 1 h at 50 °C. The reaction was quenched by the addition of an ice-cold solution of saturated NaHCO_3_ (100 mL), extracted with EtOAc (3 × 50 mL), the combined organic layers were dried (Na_2_SO_4_) and evaporated. The residue was dissolved in MeOH (10 mL) and a few drops of water were added. After several hours, the crystals formed were filtered off (ca. 1.1 g, virtually pure *trans*-isomer) and the mother liquor was evaporated (0.84 g, ca. 30% of *cis*-isomer, according to HPLC). The evaporated residue was then dissolved in a mixture of acetone/vinyl acetate (100 mL, 9:1, v/v), Novozym 435 (0.84 g, ≥10000 U/g, 100% w/w) was added, and the mixture was shaken at 45 °C and 650 rpm for 48 h. After enzyme removal by filtration, the solution was evaporated and the crude mixture was purified by column chromatography (CHCl_3_/acetone/HCO_2_H 95:5:1, twice) yielding **2a** (0.6 g, 30% yield, >98% optical purity) and **12** (0.2 g, 10% yield, >97% optical purity). ^1^H and ^13^C NMR data see Table 5. HRMS (ESI–TOF) *m*/*z*: [M − H]^−^ calcd for C_27_H_23_O_11_, 523.1246; found, 523.1245.

**(2*R*,3*S*,10*S,*11*S*)*-*23-*O*-Acetylsilybin (11) – Method A:** This compound was prepared analogously to **12** starting from pure **1b** (2 g, 4.146 mmol, >99% optical purity) to yield 343 mg of **11** (17% yield, >97% optical purity). ^1^H and ^13^C NMR data, see Table 5. HRMS (ESI–TOF) *m*/*z*: [M − H]^−^ calcd for C_27_H_23_O_11_, 523.1246; found, 523.1247.

**Table 5:** NMR spectroscopic data (600 MHz, DMSO-*d*_6_, 30 °C) of compounds **12** and **11**.

	(2*R*,3*S*,10*R,*11*R*)*-*23-*O*-acetylsilybin (**12**)			(2*R*,3*S*,10*S,*11*S*)*-*23-*O*-acetylsilybin (**11**)		
	
position	δ_C_	m^a^	δ_H_ (m, *J* in Hz)	δ_C_	m^a^	δ_H_ (m, *J* in Hz)

2	80.50	d	5.460 (d, 2.4)	80.52	d	5.461 (d, 2.4)
3	70.78	d	4.109 (br.d, 2.4, 6.4)	70.79	d	4.113 (dd, 2.4, 6.4)
4	195.29	s	–	195.31	s	–
4a	100.27	s	–	100.29	s	–
5	164.00	s	–	164.01	s	–
6	96.04	d	5.908 (d, 2.1)	96.05	d	5.912 (d, 2.1)
7	166.86	s	–	166.85	s	–
8	95.05	d	5.936 (d, 2.1)	95.05	d	5.940 (d, 2.1)
8a	162.49	s	–	162.50	s	–
10	75.01	d	4.491 (ddd, 8.0, 5.2, 2.7)	75.02	d	4.492 (ddd, 7.9, 5.2, 2.7)
11	75.85	d	4.913 (d, 8.0)	75.86	d	4.915 (d, 7.9)
12a	143.06	s	–	143.07	s	–
13	116.26	d	7.080 (d, 2.0)	116.27	d	7.083 (d, 2.0)
14	129.51	s	–	129.52	s	–
15	120.70	d	7.020 (dd, 8.4, 2.0)	120.70	d	7.022 (dd, 8.3, 2.0)
16	116.30	d	6.976 (d, 8.4)	116.31	d	6.978 (d, 8.3)
16a	142.59	s	–	142.60	s	–
17	126.67	s	–	126.68	s	–
18	111.74	d	7.013 (d, 1.9)	111.74	d	7.014 (d, 2.0)
19	147.80	s	–	147.81	s	–
20	147.34	s	–	147.34	s	–
21	115.50	d	6.809 (d, 8.0)	115.51	d	6.812 (d, 8.0)
22	120.60	d	6.861 (dd, 8.0, 1.9)	120.61	d	6.863 (dd, 2.0, 8.0)
23	62.65	t	4.081 (dd, 2.7, 12.4)	62.66	m	4.084 (dd, 2.7, 12.4)
			3.926 (dd, 5.2, 12.4)		d	3.928 (dd, 5.2 , 12.4)
3-OH	–	–	6.223 (d, 6.4)	–	d	6.227 (d, 6.4)
5-OH	–	–	11.861 (s)	–	s	11.863 (s)
7-OH	–	–	10.805 (br s)	–	s	10.813 (br s)
19-OMe	55.75	q	3.773 (s)	55.75	s	3.775 (s)
20-OH	–	–	9.164 (s)	–	d	9.167 (s)
23-C=O	170.06	s	–	170.07	s	–
23-Ac	20.46	q	2.021 (s)	20.47	m	2.022 (s)

^a^multiplicity of ^13^C signals.

**(2*R*,3*S*,10*S,*11*S*)*-*23-*O*-Acetylsilybin (11) – Method B:** BF_3_∙OEt_2_ (2.3 mL, 18.3 mmol, 50% solution in OEt_2_) was added to a stirred solution of **1b** (3 g, 6.2 mmol, >99% optical purity) in EtOAc (100 mL) and the mixture was stirred for 3 h at 80 °C. The reaction mixture was quenched by the addition of an ice-cold solution of saturated NaHCO_3_ (100 mL), and after stirring for 10 min both phases were separated. The aqueous phase was extracted with EtOAc (3 × 50 mL), the combined organic layers were dried (Na_2_SO_4_) and evaporated. The residue after flash chromatography (CHCl_3_/acetone/HCO_2_H, 97:3:1, twice) yielded title compound **11** (50 mg, 2% yield, >98% optical purity). ^1^H and ^13^C NMR data, see Table 5. HRMS (ESI–TOF) *m/z*: [M − H]^−^ calcd. for C_27_H_23_O_11_, 523.1246; found, 523.1246.

**(2*R*,3*S*,10*R,*11*R*)*-*Silybin (9):** Novozym 435 (1 g, ≥10000 U/g) was added to a solution of **12** (200 mg, 0.381 mmol) in a mixture of MTBE/*n*-butanol (30 mL, 9:1, v/v), and the mixture was shaken at 45 °C and 650 rpm for 100 h. The enzyme was removed by filtration, the solution was evaporated, and the crude mixture purified by column chromatography (CHCl_3_/acetone/HCO_2_H from 95:5:1 to 70:30:1) yielding title compound **9** (140 mg, 70% yield, >97% purity). ^1^H and ^13^C NMR data, see Table 6. [α]_D_^22^ −51.6 (*c* 0.091, acetone); ECD spectrum, see Supporting Information File 1, Figure S2; HRMS (ESI–TOF) *m*/*z*: [M + H]^+^ calcd for C_25_H_23_O_10_, 483.1291; found, 483.1296.

**(2*R*,3*S*,10*S,*11*S*)*-*Silybin (10):** This compound was prepared analogously to **9** starting from pure **11** (343 mg, 0.654 mmol) to yield **8** (220 mg, 64% yield, >96% purity). ^1^H and ^13^C NMR data, see Table 6. [α]_D_^22^ −40.4 (*c* 0.39, acetone); ECD spectrum, see Supporting Information File 1, Figure S1; HRMS (ESI–TOF) *m*/*z*: [M + H]^+^ calcd. for C_25_H_23_O_10_, 483.1291; found, 483.1294.

**Table 6:** NMR spectroscopic data (600 MHz, DMSO-*d*_6_, 30 °C) of compounds **9** and **10**.

	(2*R*,3*S*,10*R,*11*R*)*-*silybin (**9**)			(2*R*,3*S*,10*S,*11*S*)*-*silybin (**10**)		
	
position	δ_C_	m^a^	δ_H_ (m, *J* in Hz)	δ_C_	m^a^	δ_H_ (m, *J* in Hz)

2	80.60	d	5.441 (ddd, 2.4, 0.7, 0.6)	80.60	d	5.443 (ddd, 2.5, 0.6, 0.4)
3	70.81	d	4.092 (br.d, 2.4)	70.82	d	4.087 (m, -)
4	195.21	s	–	195.25	s	–
4a	100.17	s	–	100.22	s	–
5	164.03	s	–	164.04	s	–
6	96.08	d	5.897 (d, 2.1)	96.07	d	5.903 (d, 2.1)
7	167.15	s	–	167.04	s	–
8	95.09	d	5.925 (d, 2.1)	95.08	d	5.934 (d, 2.1)
8a	162.54	s	–	162.57	s	–
10	78.14	d	4.151 (ddd, 7.9, 4.7, 2.5)	78.15	d	4.144 (ddd, 7.8, 4.7, 2.6)
11	75.86	d	4.889 (d, 7.9)	75.90	d	4.891 (d, 7.8)
12a	143.11	s	–	143.21	s	–
13	116.14	d	7.055 (dd, 2.0, 0.7)	116.15	d	7.052 (dd, 2.0, 0.4)
14	129.09	s	–	129.07	s	–
15	120.46	d	6.998 (ddd, 8.3, 2.0, 0.6)	120.47	d	7.001 (ddd, 8.3, 2.0, 0.6)
16	116.18	d	6.945 (d, 8.3)	116.19	d	6.947 (d, 8.3)
16a	143.19	s	–	143.11	s	–
17	127.56	s	–	127.57	s	–
18	111.76	d	7.002 (d, 2.0)	111.79	d	7.003 (d, 1.9)
19	147.67	s	–	147.68	s	–
20	147.06	s	–	147.07	s	–
21	115.37	d	6.802 (d, 8.1)	115.38	d	6.803 (d, 8.1)
22	120.54	d	6.858 (dd, 8.1, 2.0)	120.55	d	6.860 (dd, 1.9, 8.1)
23	60.23	t	3.534 (dd, 2.5, 12.2)	60.24	t	3.532 (m)
			3.341 (dd, 4.7, 12.2)			3.340 (m)
3-OH	–	–	6.216 (br.s)		–	6.212 (br.d, 5.5)
5-OH	–	–	11.884 (br.s)		–	11.881 (s)
7-OH	–	–	n.d.		–	n.d.
19-OMe	55.75	q	3.777 (s)	55.76	q	3.780 (s)
20-OH	–	–	9.151 (br.s)		–	9.118 (br.s)
23-OH	–	–			d	

^a^multiplicity of ^13^C signals.

**(2*R*,3*R*,10*S,*11*R*)*-*23-*O*-Acetylsilybin (8):** BF_3_∙OEt_2_ (1.5 mL, 12.2 mmol, 50% solution in OEt_2_) was added to a stirred solution of **1b** (2 g, 4.1 mmol, >99% optical purity) in EtOAc (100 mL) and the mixture was kept for 48 h at 80 °C. The reaction mixture was quenched by the addition of an ice-cold solution of saturated NaHCO_3_ (100 mL), and after stirring for 10 min both phases were separated. The aqueous phase was extracted with EtOAc (3 × 50 mL). The combined organic layers were dried (Na_2_SO_4_) and evaporated to dryness. The crude mixture was purified by column chromatography (CHCl_3_/acetone/toluene/HCO_2_H, 95:5:5:1, twice) yielding a mixture of **2b** and **8**, which was then dissolved in a mixture of MTBE/*n*-butanol (150 mL, 9:1 v/v), Novozym 435 (0.25 g, ≥10000 U/g, 100% w/w) was added, and the mixture was shaken at 45 °C and 650 rpm for 37 h until the ratio of **8**/**2b** was 96:4 (HPLC). After enzyme removal by filtration, the solution was evaporated, and the crude mixture purified by column chromatography (CHCl_3_/acetone/HCO_2_H 90:10:1) yielding **1b** (0.898 g, 45% yield, >99% purity) and **12** (0.06 g, 3% yield, >98% purity). ^1^H and ^13^C NMR data, see Table 7. HRMS (ESI–TOF) *m*/*z*: [M − H]^−^ calcd for C_27_H_23_O_11_, 523.1246; found, 523.1244.

**(2*S*,3*S*,10*R,*11*S*)*-*23-*O*-Acetylsilybin (13):** BF_3_∙OEt_2_ (2.7 mL, 21.9 mmol, 50% solution in OEt_2_) was added to a stirred solution of **1a** (2.7 g, 5.6 mmol, >99% optical purity) in EtOAc (30 mL) and the mixture was kept at 80 °C for 48 h. The reaction mixture was quenched by the addition of an ice-cold solution of saturated NaHCO_3_ (100 mL), and after stirring for 10 min both phases were separated. The aqueous phase was extracted with EtOAc (3 × 50 mL), the combined organic layers were dried (Na_2_SO_4_) and evaporated. The crude mixture was purified by column chromatography (CHCl_3_/acetone/HCO_2_H 95:5:1, twice), yielding **12** (0.32 g; 12%, >96% purity) and a mixture of **2a** and **13**. After evaporation of the solvent, the residue containing compounds **2a** and **13** (1 g) was then dissolved in a mixture of MTBE/*n*-butanol (30 mL, 9:1, v/v), Novozym 435 (0.3 g, ≥10000 U/g, 30% w/w) was added and the mixture was shaken at 45 °C and 650 rpm for 72 h to give a **13**/**2a** ratio of ca. 2:3 (HPLC). After enzyme removal by filtration, the solution was evaporated, and the crude mixture was purified by column chromatography (CHCl_3_/acetone/HCO_2_H 90:10:1) to remove **1a**. Subsequent preparative HPLC separation (A Chromolith SemiPrep RP-18e monolithic column, 100 × 10 mm, Merck) of the mixture of **2a** and **13** was carried out with an isocratic mobile phase CH_3_OH/H_2_O 50:50, a flow rate of 5 mL/min at 25 °C, and UV detection at 285 nm; *t*_r_ (**2a**) = 6.6 min, *t*_r_ (**13**) = 7.8 min.) yielding **2a** (20 mg, 0.7% yield, >98% purity) and **13** (7 mg, 0.3% yield, >96% purity). [α]_D_^22^ +69.7 (*c* 0.11, acetone); ECD spectrum, see Supporting Information File 1, Figure S4; ^1^H and ^13^C NMR data, see Table 7; HRMS (ESI–TOF) *m*/*z*: [M + Na]^+^ calcd for C_27_H_24_O_11_Na, 547.1211; found, 547.1209.

**Table 7:** NMR spectroscopic data (600 MHz, DMSO-*d*_6_, 30 °C) of compounds **8** and **13**.

	(2*R*,3*R*,10*S,*11*R*)*-*23-*O*-acetylsilybin (**8**)			(2*S*,3*S*,10*R,*11*S*)*-*23-*O*-acetylsilybin (**13**)		
	
position	δ_C_	m^a^	δ_H_ (m, *J* in Hz)	δ_C_	m^a^	δ_H_ (m, *J* in Hz)

2	82.48	d	5.101 (d, 11.4)	82.44	d	5.085 (dd, 0.4, 11.3)
3	71.46	d	4.618 (dd, 6.1, 11.4)	71.46	d	4.597 (dd, 6.3, 11.3)
4	197.71	s	–	197.32	s	–
4a	100.48	s	–	100.21	s	–
5	163.32	s	–	163.36	s	–
6	96.13	d	5.923 (d, 2.1)	96.31	d	5.879 (br.s, –)
7	166.86	s	–	167.00	s	–
8	95.07	d	5.883 (d, 2.1)	95.31	d	5.848 (br.s, –)
8a	162.48	s	–	162.44	s	–
10	73.83	d	4.801 (ddd, 2.8, 3.4, 8.2)	73.87	d	4.793 (ddd, 2.9, 3.4, 8.3)
11	74.60	d	5.340 (dd, 0.6, 2.8)	74.60	d	5.337 (ddd, 0.4, 0.7, 2.9)
12a	142.43	s	–	142.47	s	–
13	116.95	d	7.167 (d, 2.0)	116.87	d	7.171 (d, 2.0)
14	130.86	s	–	130.95	s	–
15	121.79	d	7.066 (dd, 2.0, 8.3)	121.97	d	7.049 (ddd, 0.4, 2.0, 8.3)
16	116.92	d	7.013 (d, 8.3)	116.93	d	7.005 (d, 8.3)
16a	141.73	s	–	141.70	s	–
17	126.52	s	–	126.53	s	–
18	110.73	d	7.023 (d, 2.0)	110.65	d	7.026 (dd, 0.4, 2.1)
19	147.62	s	–	147.63	s	–
20	146.65	s	–	146.65	s	–
21	115.59	d	6.811 (d, 8.2)	115.59	d	6.807 (d, 8.1)
22	118.67	d	6.860 (ddd, 0.6, 2.0, 8.2)	118.66	d	6.859 (ddd, 0.7, 2.1, 8.1)
23	60.38	t	4.004 (dd, 3.4, 12.2)	55.73	t	3.998 (dd, 3.4, 12.2)
			3.954 (dd, 8.2, 12.2)			3.995 (dd, 8.3, 12.2)
3-OH	–	–	5.783 (d, 6.1)	–		5.769 (d, 6.3)
5-OH	–	–	11.875 (s)	–		11.902 (s, –)
7-OH	–	–	10.788 (br s)	–		10.860 (br.s, –)
19-OMe	55.73	q	3.757 (s)	55.73	q	3.757 (s, –)
20-OH	–	–	9.093 (s)	–		9.119 (s, –)
23-CO	170.08	s	–	170.13	s	–
23-Ac	20.46	q	1.941 (s)	20.51	q	1.940 (s, –)

^a^multiplicity of ^13^C signals.

**(2*R*,3*R*,10*S,*11*R*)*-*Silybin (14):** Novozym 435 (0.350 g, 500% w/w, ≥10000 U/g) was added to **12** (60 mg, 0.11 mmol) dissolved in a mixture of MTBE/*n*-butanol (15 mL, 9:1, v/v), and the mixture was shaken at 45 °C and 650 rpm for 120 h. The enzyme was then filtered off, the solution evaporated, and the crude mixture purified by column chromatography (CHCl_3_/acetone/HCO_2_H from 97:3:1 to 70:30:1) yielding title compound **14** (40 mg, 67% yield, >98% purity). [α]_D_^22^ −59.2 (*c* 0.13, acetone); ECD spectrum, see Supporting Information File 1, Figure S3; ^1^H and ^13^C NMR data, see Table 8; HRMS (ESI–TOF) *m*/*z*: [M + H]^+^ calcd for C_25_H_23_O_10_, 483.1291; found, 483.1289

**Table 8:** NMR spectroscopic data (600 MHz, DMSO-*d*_6_, 30 °C) of compound **14**.

	(2*R*,3*R*,10*S,*11*R*)*-*silybin (**14**)		

position	δ_C_ [ppm]	m^a^	δ_H_ (m, *J* in Hz)

2	82.56	d	5.082 (dd, 0.4, 11.4)
3	71.44	d	4.608 (dd, 5.8, 11.4)
4	197.69	s	–
4a	100.43	s	–
5	163.30	s	–
6	96.12	d	5.913 (d, 2.1)
7	166.94	s	–
8	95.08	d	5.873 (d, 2.1)
8a	162.49	s	–
10	77.36	d	4.464 (ddd, 2.8, 4.0, 7.9)
11	75.13	d	5.272 (ddd, 0.5, 0.5, 2.8)
12a	142.69	s	–
13	116.74	d	7.106 (d, 2.0)
14	130.53	s	–
15	121.36	d	7.037 (ddd, 0.4, 2.0, 8.3)
16	116.85	d	6.986 (d, 8.3)
16a	142.47	s	–
17	127.23	s	–
18	111.21	d	6.966 (dd, 0.5, 1.8)
19	147.44	s	–
20	146.49	s	–
21	115.44	d	6.770 (d, 8.2)
22	119.05	d	6.791 (ddd, 0.5, 1.8, 8.2)
23	58.20	t	3.409 (dd, 7.9, 11.6)
			3.338 (dd, 4.0, 11.6)
3-OH	–	–	5.766 (d, 5.8)
5-OH	–	–	11.876 (s, –)
7-OH	–	–	10.490 (br.s)
19-OMe	55.72	q	3.732 (s, –)
20-OH	–	–	9.032 (s, –)
23-OH	–	–	4.833 (br.s)

^a^multiplicity of ^13^C signals.

### Calculation methods

All geometries and energies, including the zero-point correction (V), enthalpies (*H*) and Gibbs energies (*G*) at 298 K of the reactants, intermediates and products were determined at the (U)B3P86/6-31+G(d,p) level, well-adapted for polyphenol reactivity. Solvent effects were implicitly taken into account by using a PCM (polarizable continuum model) method; the IEFPCM (integral equation formalism PCM) method coupled to UA0 radii was used. The mechanisms were studied with both EtOAc and DMF solvents with a dielectric constant ε of 5.99 and 37.21, respectively.

The hardness η is related to the hard–soft-acid-basis (HSAB) principle. According to this theory, hard acids react with hard bases whereas soft acids react with soft bases. The hardness is given by 
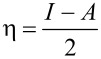
 where *I* and *A* are the adiabatic ionization potential and the adiabatic electron affinity, respectively. The hardness of silybin B was not calculated, as the stereochemistry is not expected to significantly modify this parameter. The hardness calculation is based on the HOMO (highest occupied molecular orbiral) and LUMO (lowest occupied molecular orbital) energies, which are the identical for silybin A and B. The hardness was calculated in benzene, EtOAc, and DMF to study the impact of the solvent polarity on the HSAB principle. The hardness of BF_3_ and silybin A is not significantly modified when the solvent polarity is increased (Table 2).

New methods to rationalize chemical reactivity have been developed in the field of quantum mechanical methods over the past few years. The Fukui function *f_k_(r)* has become one of these powerful tools, providing an atomic picture of hardness. For a given atom *k*, it is given by 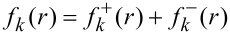 where *f_k_^+^*(r) and *f*_k_^−^(r) are the electrophilic and nucleophilic contributions of the Fukui function calculated as follows:


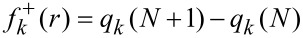


and 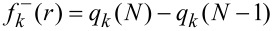, where *q_k_*(*N*), *q_k_*(*N−*1) and *q_k_*(*N* + 1) are the electronic population of atom *k* in its neutral, radical-cation and radical-anion forms, respectively. In this study, the Fukui function is used to partially rationalize BF_3_ complexation. In this case, the nucleophilic contribution is the most important parameter. It must be stressed that the higher the *f_k_*^−^(r), the higher the atomic nucleophilic capacity. All calculations were carried out by using the Gaussian09 software [35].

## Supporting Information

File 1 ^1^H and ^13^C NMR spectra of new compounds, ECD spectra of new compounds, HPLC chromatograms of new compounds, table of retention times and purity of the new compounds, XYZ coordinates of optimized silybin A and B and absolute energies.
