# Supplementary material for: cis–trans Isomerization of silybins A and B
Source: Beilstein J Org Chem. 2014 May 8;10:1047–63. doi: 10.3762/bjoc.10.105 (PMC4077359; doi:10.3762/bjoc.10.105)
Supplement: File 1 — 1H and 13C NMR spectra of new compounds, ECD spectra of new compounds, HPLC chromatograms of new compounds, table of retention times and purity of the new compounds, XYZ coordinates of optimized silybin A and B and absolute energies. [file Beilstein_J_Org_Chem-10-1047-s001.pdf]

Supporting Information

for

cis–trans Isomerization of silybins A and B

Michaela Novotná[1§], Radek Gažák[1,2§], David Biedermann[1], Florent Di Meo[3,4], Petr Marhol[1], Marek Kuzma[1], Lucie Bednárová[5], Kateřina Fuksová[1], Patrick Trouillas[3,6,7] and Vladimír Křen[1]*

Address: [1]Institute of Microbiology, v.v.i. AS CR, Vídeňská 1083, Prague 4, CZ-14220, Czech Republic, [2]Department of Biochemistry, Faculty of Science, Charles University in Prague, Hlavova 8, CZ-12840 Prague 2, Czech Republic, [3]Inserm UMR-S850, Faculté de Pharmacie, Université de Limoges, 2 Rue du Docteur Marcland, F-87025 Limoges, France, [4]Present address: Department of Physics, Chemistry and Biology (IFM), Linköping University, SE-58183, Linköping, Sweden, [5]Institute of Organic Chemistry and Biochemistry, v.v.i. AS CR, Flemingovo náměstí 2, Prague 6, CZ-16610, Czech Republic, [6]Department of Physical Chemistry, University of Olomouc, tř. 17. listopadu 12, CZ-77146 Olomouc, Czech Republic and [7]Laboratoire de Chimie des Matériaux Nouveaux, Université de Mons, Place du Parc 20, B-7000 Mons, Belgium

Email: Vladimír Křen* - kren@biomed.cas.cz

*Corresponding author

[§]These two authors contributed equally to this work.

^{1}H and ^{13}C NMR spectra of new compounds, ECD spectra of new compounds, HPLC chromatograms of new compounds, table of retention times and purity of the new compounds, XYZ coordinates of optimized silybin A and B and absolute energies

Table of content:

ECD Spectra S3

^{1}H and ^{13}C NMR Spectra S6

HPLC Chromatograms S20

Table of retention times and purities S24

XYZ Coordinates of optimized silybin A and B S25

Absolute energies S77

Input line for optimization with Gaussian09 package S78

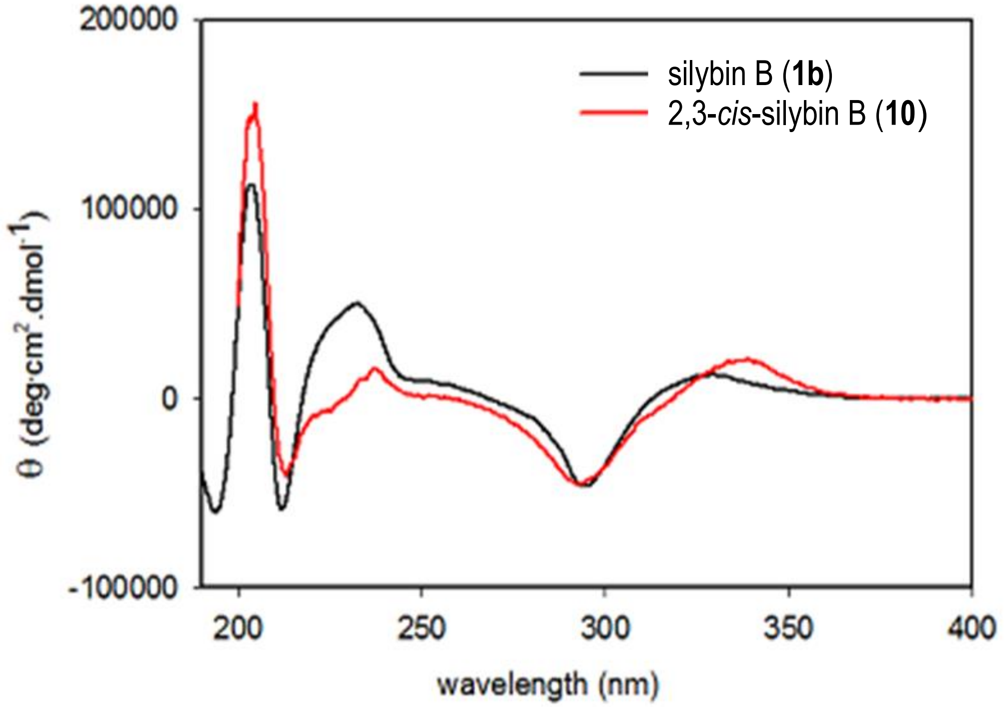

Figure S1. Comparison of ECD spectra of 2,3-*cis*-silybin B (**10**) and 2,3-*trans*-silybin B (**1b**).

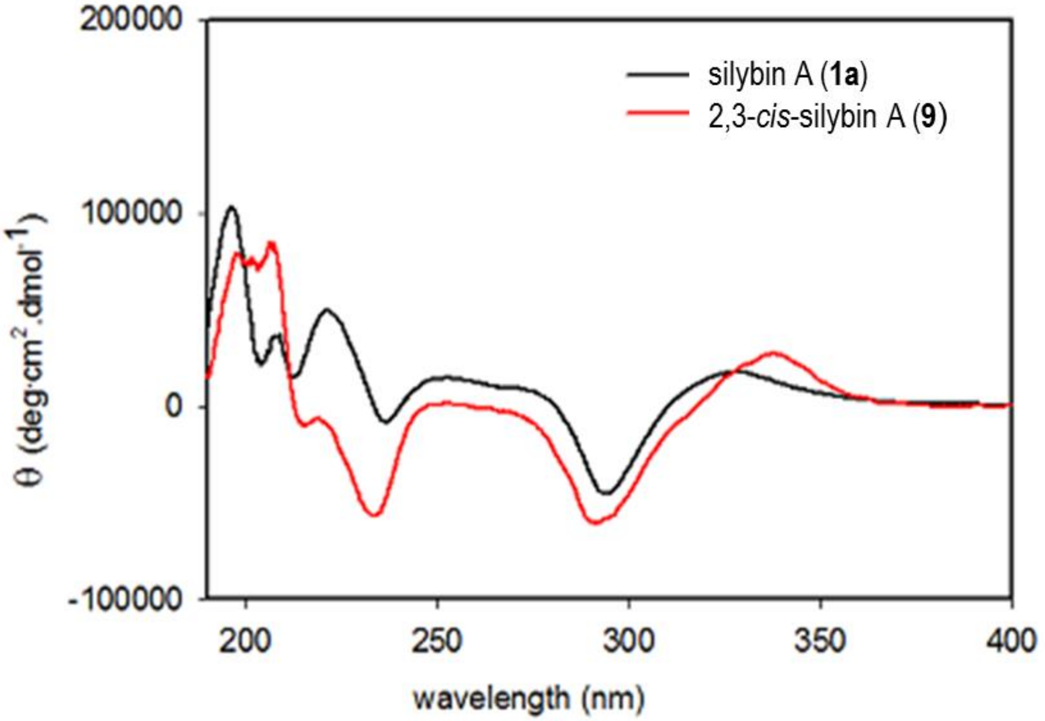

Figure S2. Comparison of ECD spectra of 2,3-*cis*-silybin A (**9**) and 2,3-*trans*-silybin A (**1a**).

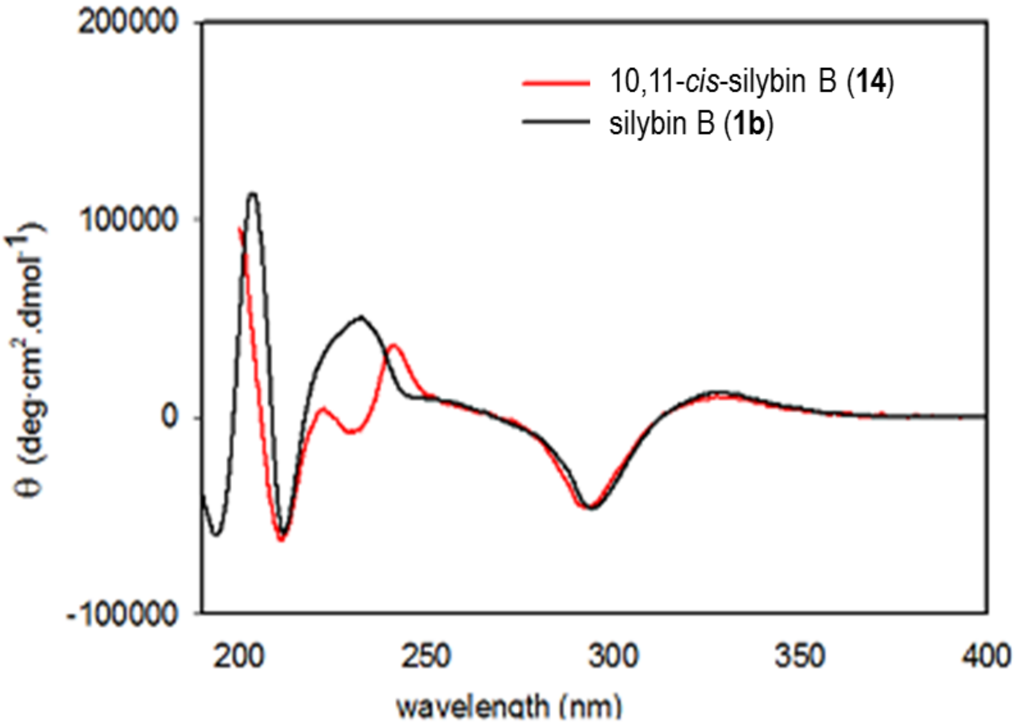

Figure S3. Comparison of ECD spectra of 10,11-*cis*-silybin B (**14**) and 10,11-*trans*-silybin B (**1b**).

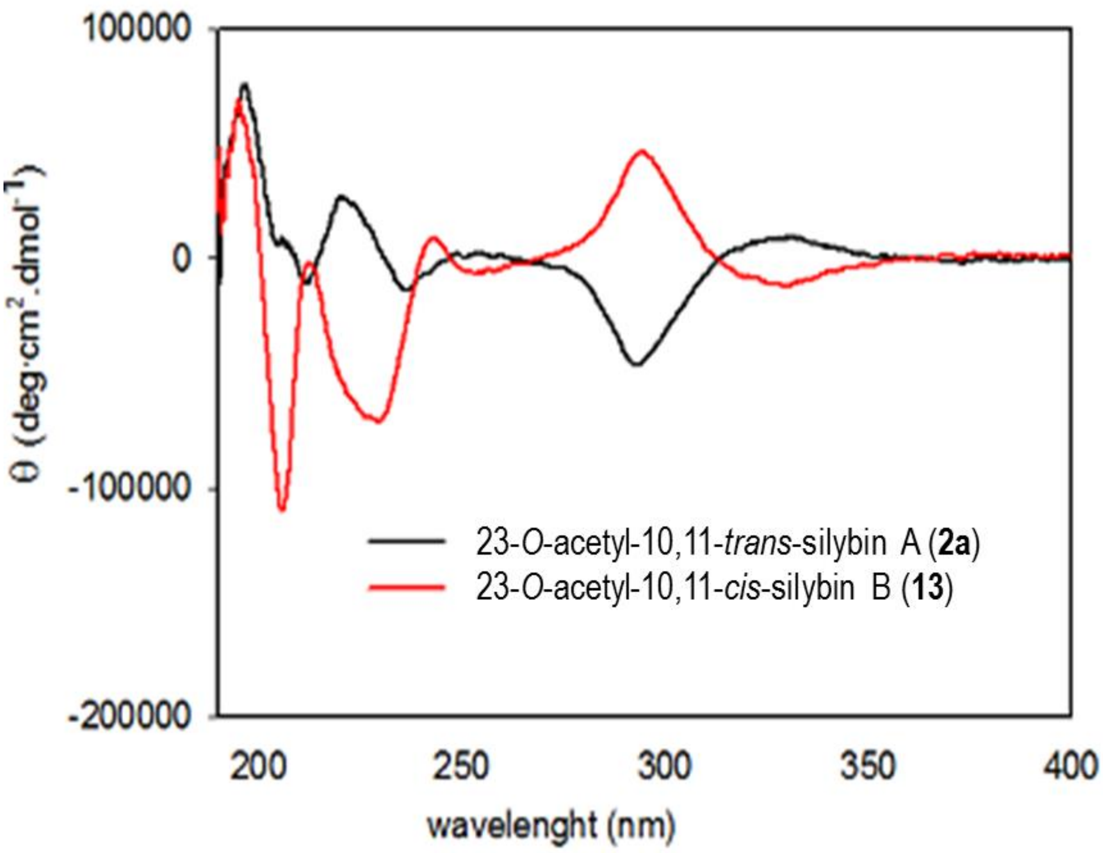

Figure S4. Comparison of ECD spectra of 23-*O*-acetyl-10,11-*cis*-silybin A (**13**) and 23-*O*-acetyl-10,11-*trans*-silybin A (**2a**).

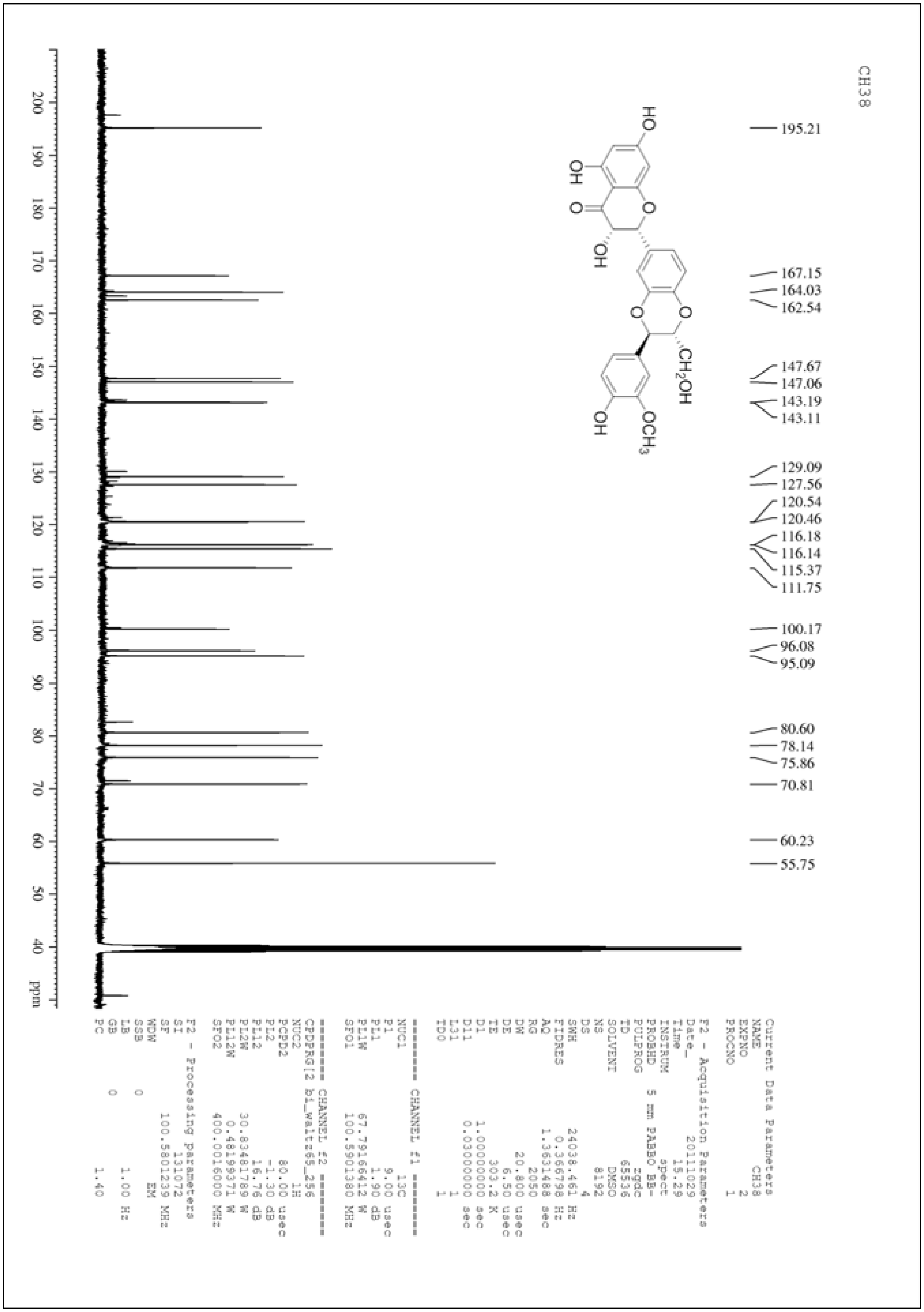

Figure S5. ^{13}C NMR Spectrum of **9**.

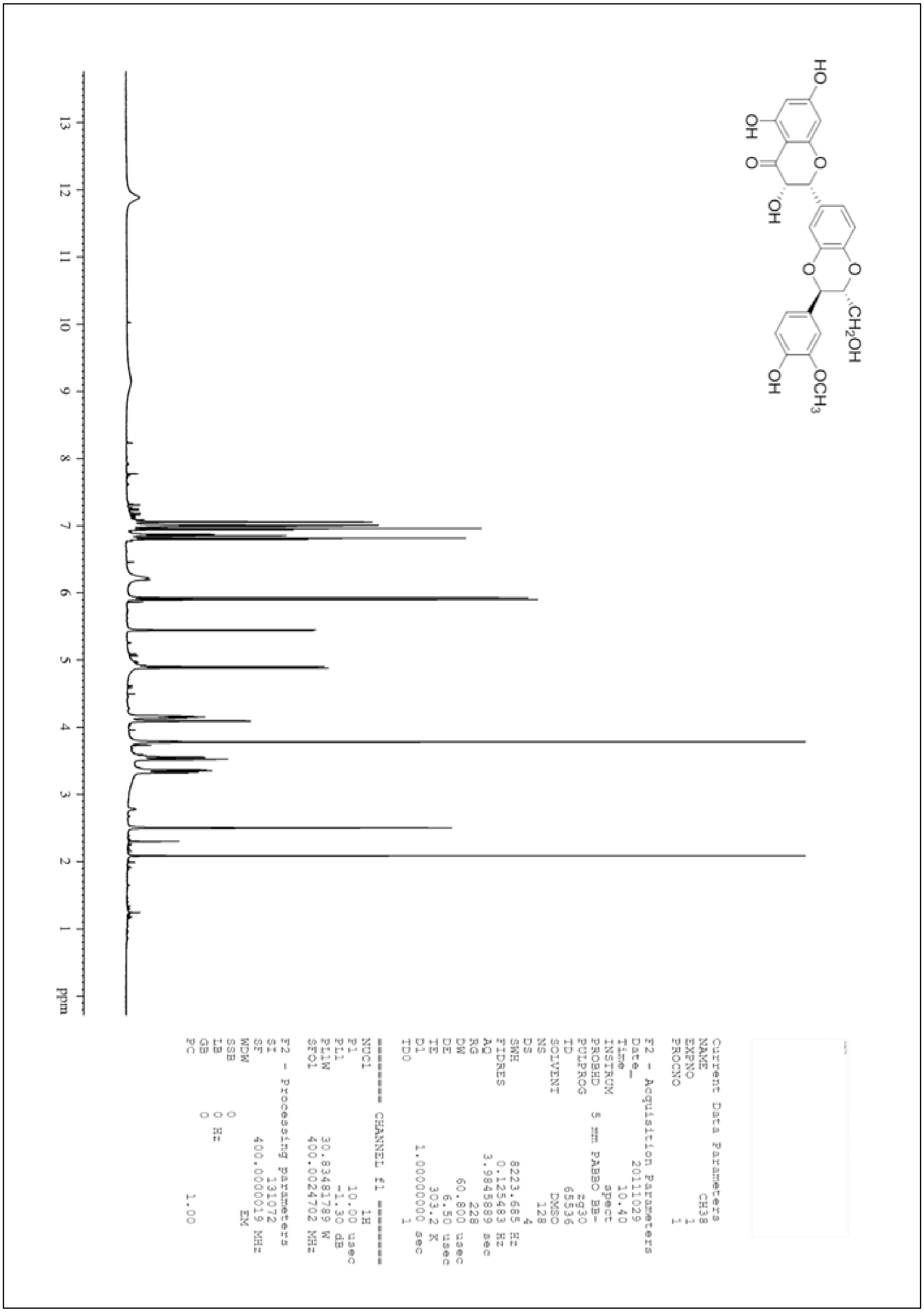

Figure S6. ^{1}H NMR Spectrum of **9**.

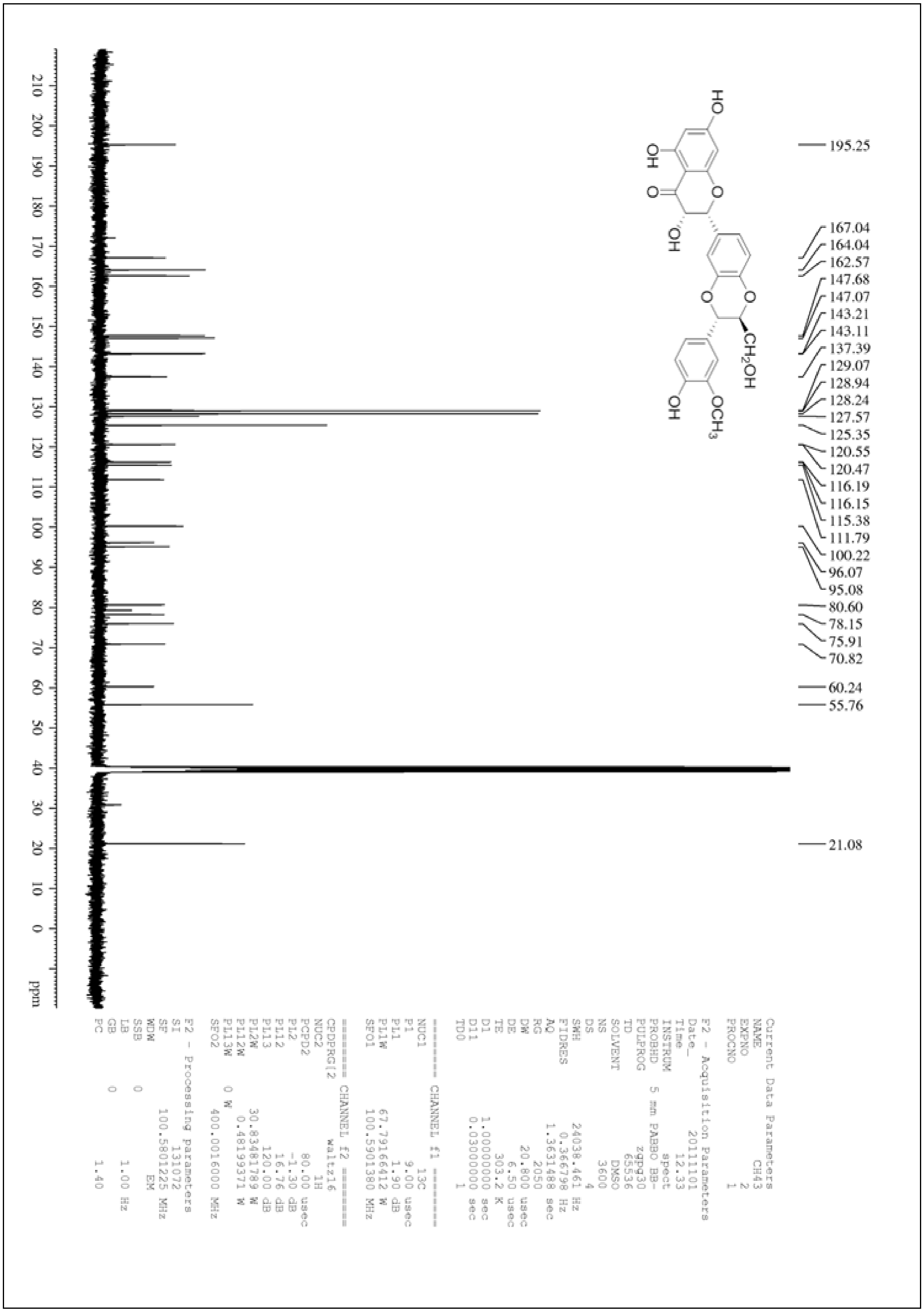

Figure S7. ^{13}C NMR Spectrum of **10**.

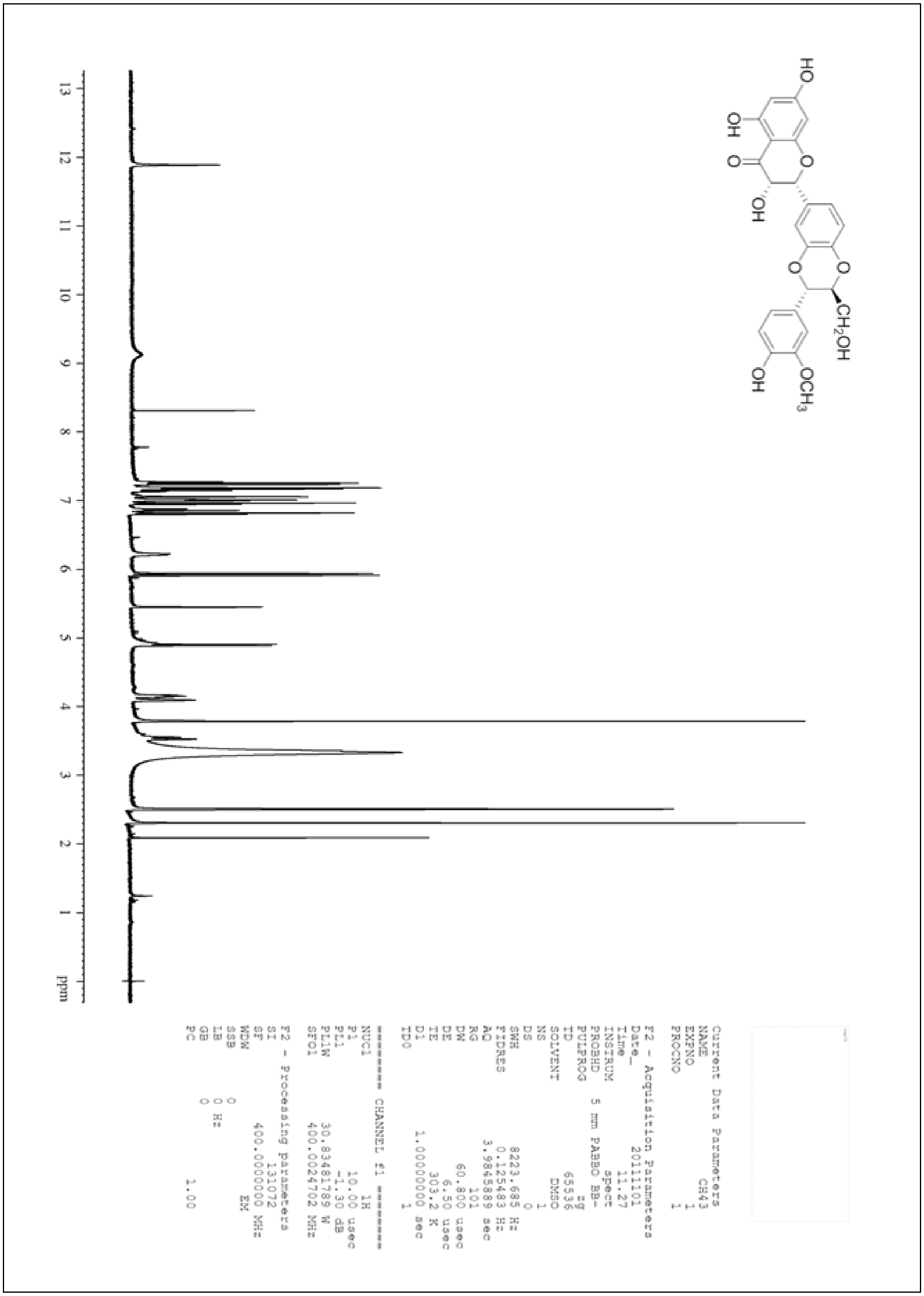

Figure S8. ^{1}H NMR Spectrum of **10**.

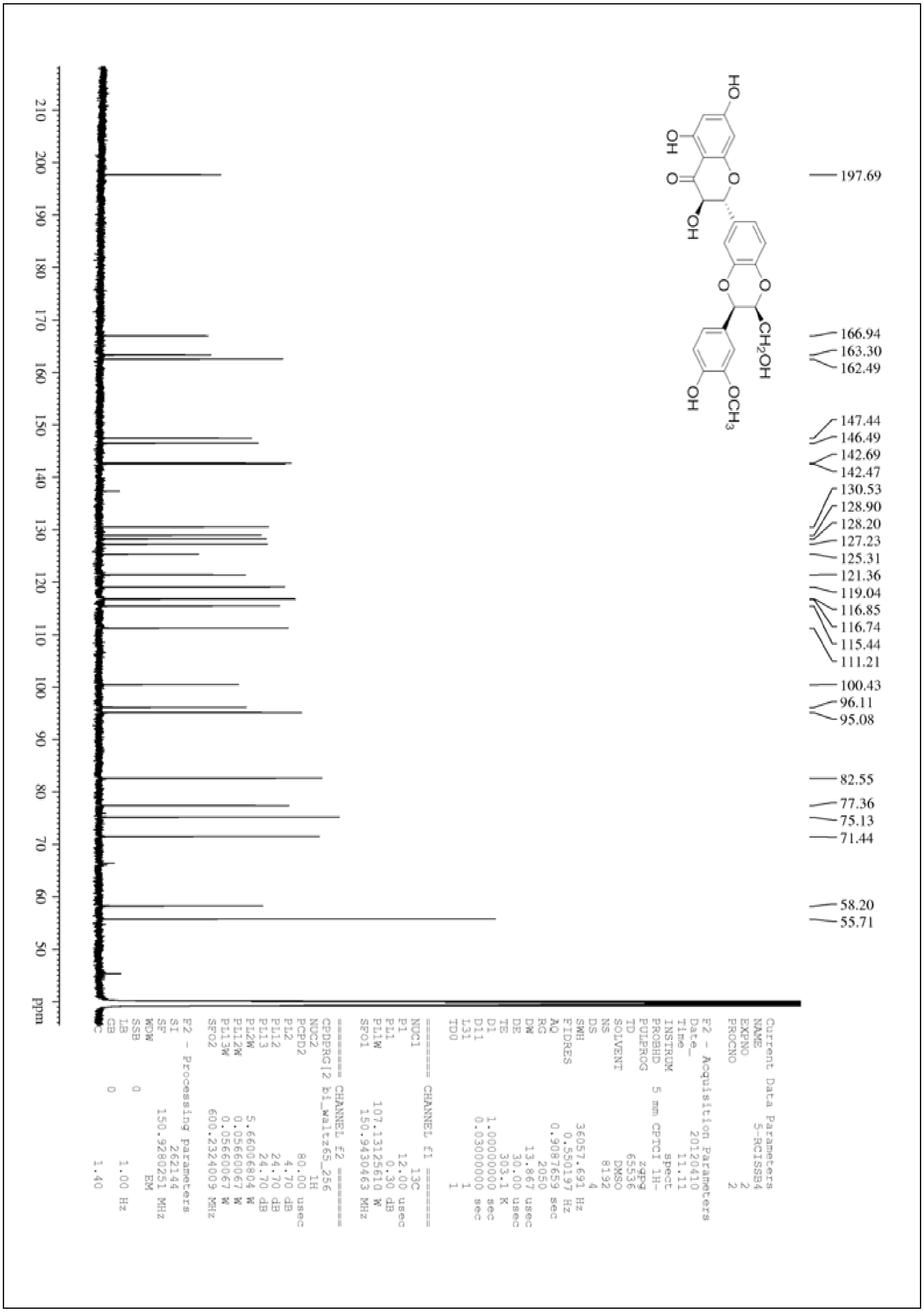

Figure S9. ^{13}C NMR Spectrum of **14**.

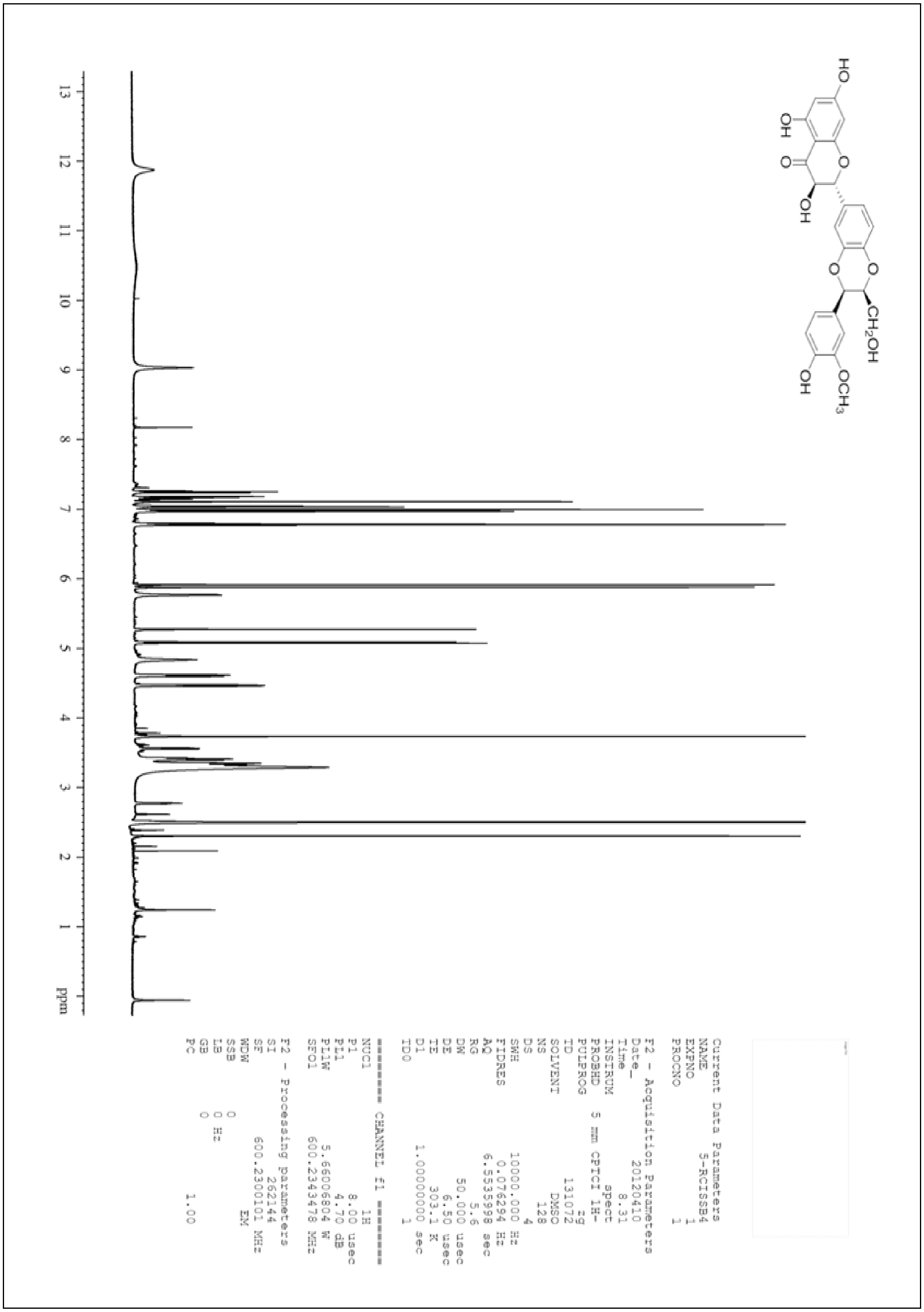

Figure S10. ^{1}H NMR Spectrum of **14**.

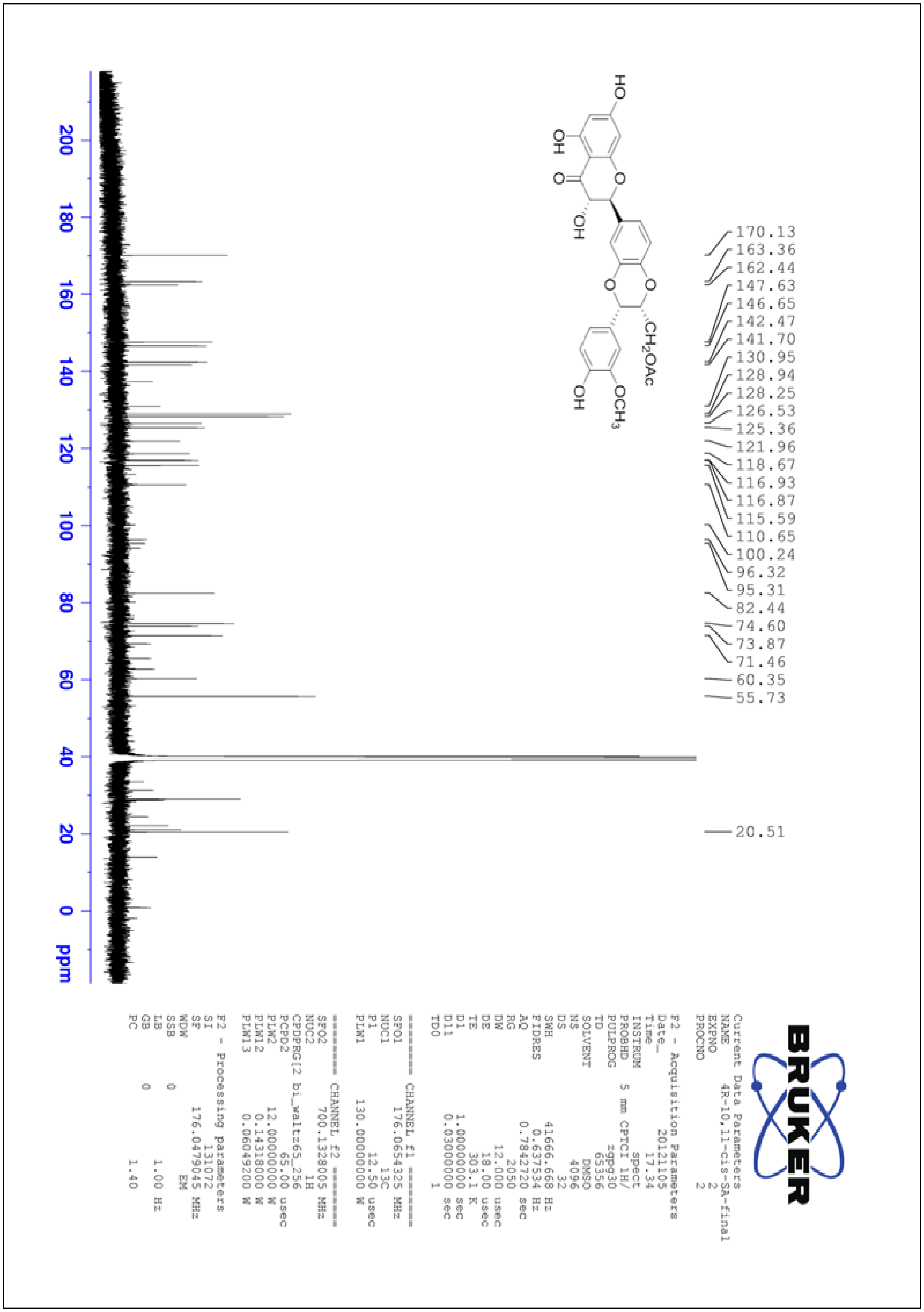

Figure S11. ^{13}C NMR Spectrum of **13**.

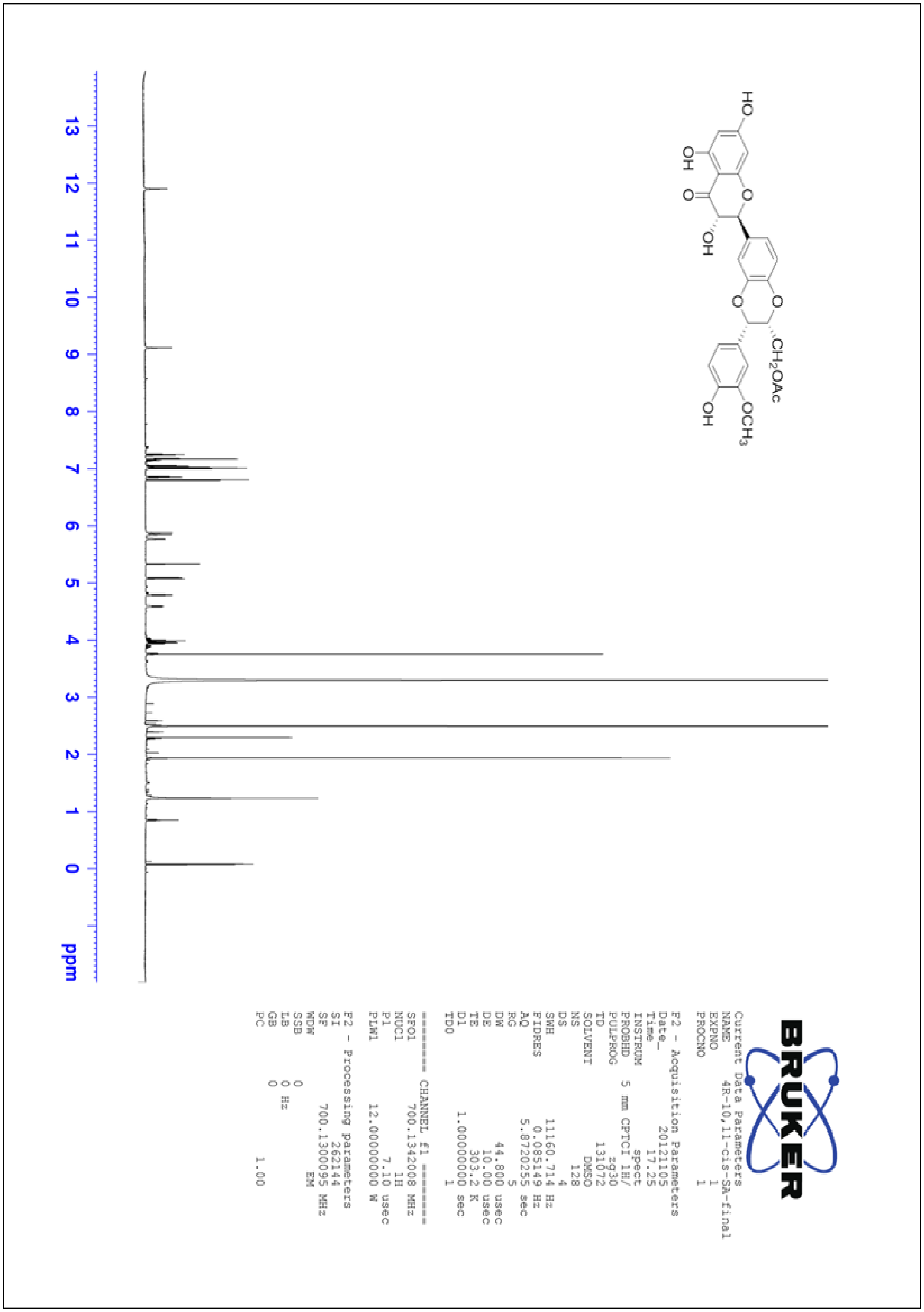

Figure S12. ^{1}H NMR Spectrum of **13**.

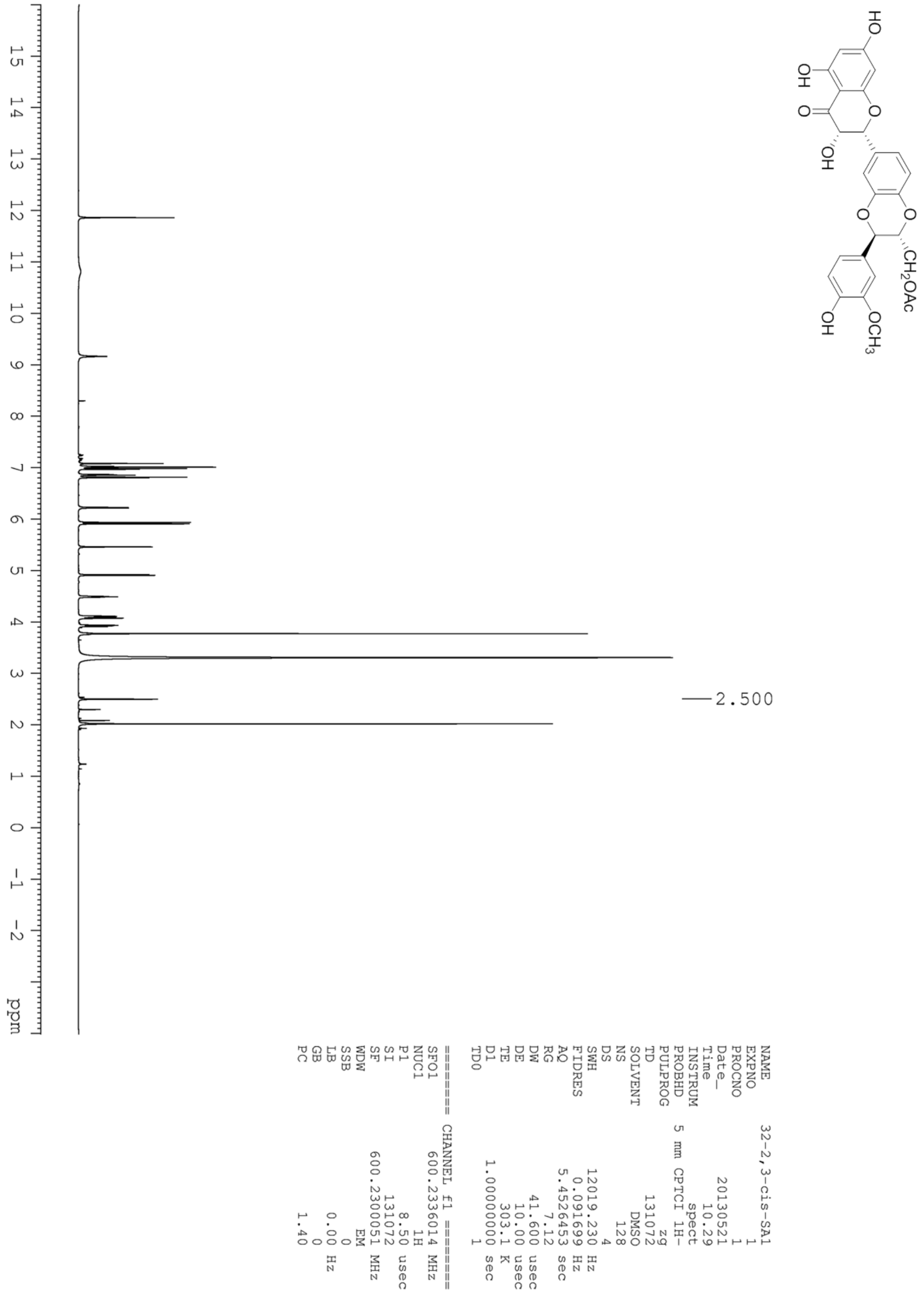

Figure S13. ^{1}H NMR Spectrum of **12**.

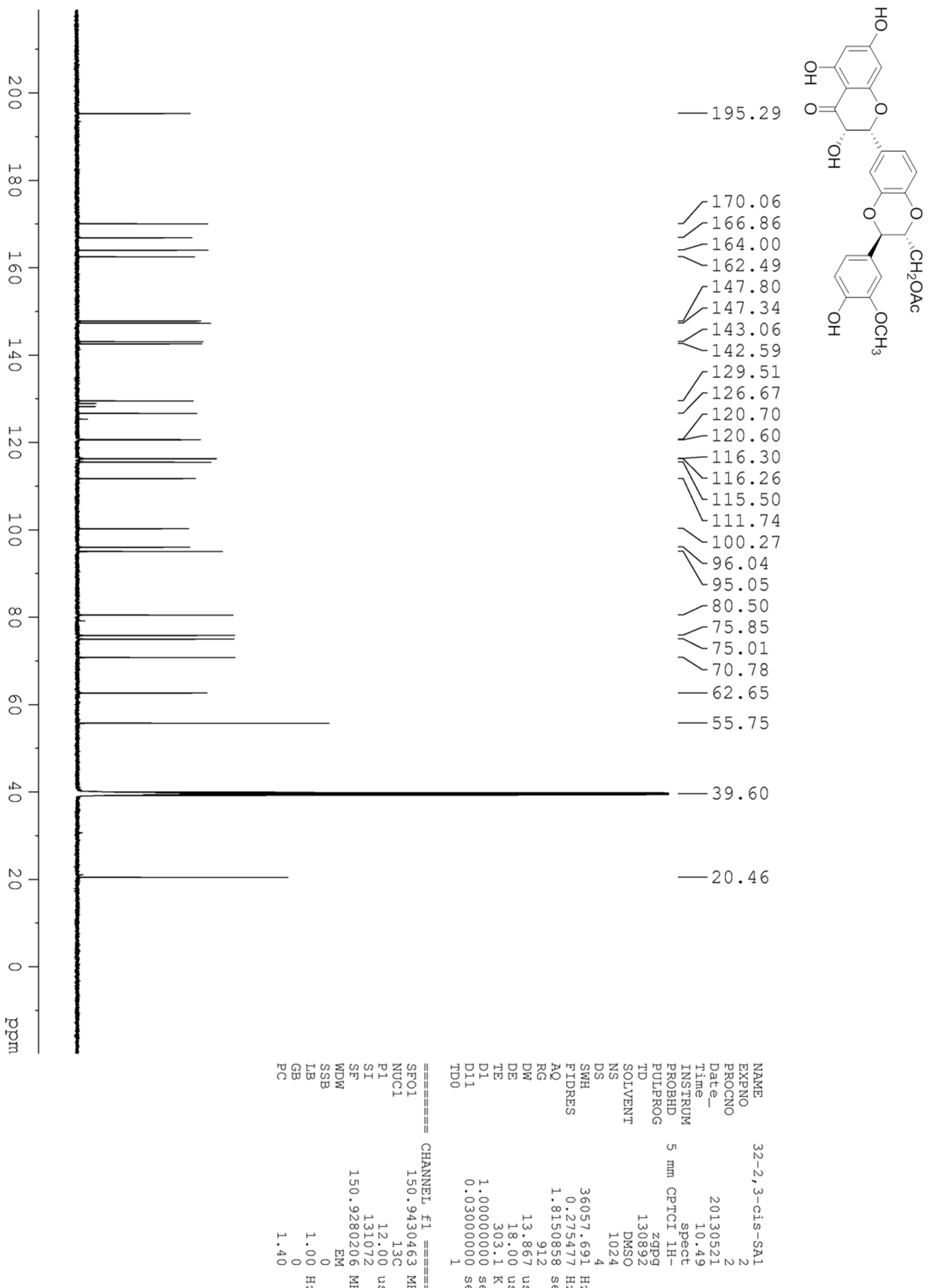

Figure S14. ^{13}C NMR Spectrum of **12**.

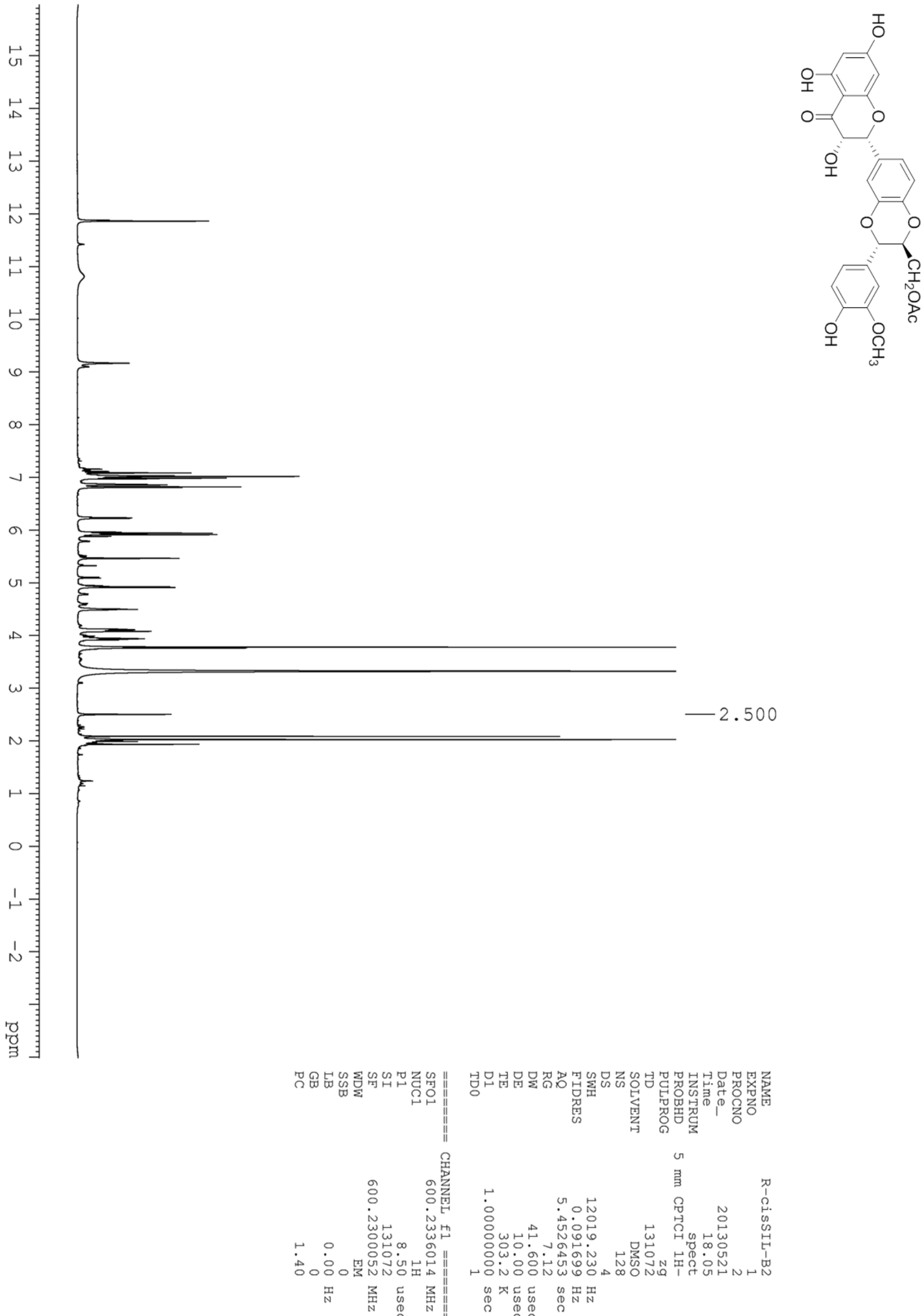

Figure S15. ^{1}H NMR Spectrum of **11**.

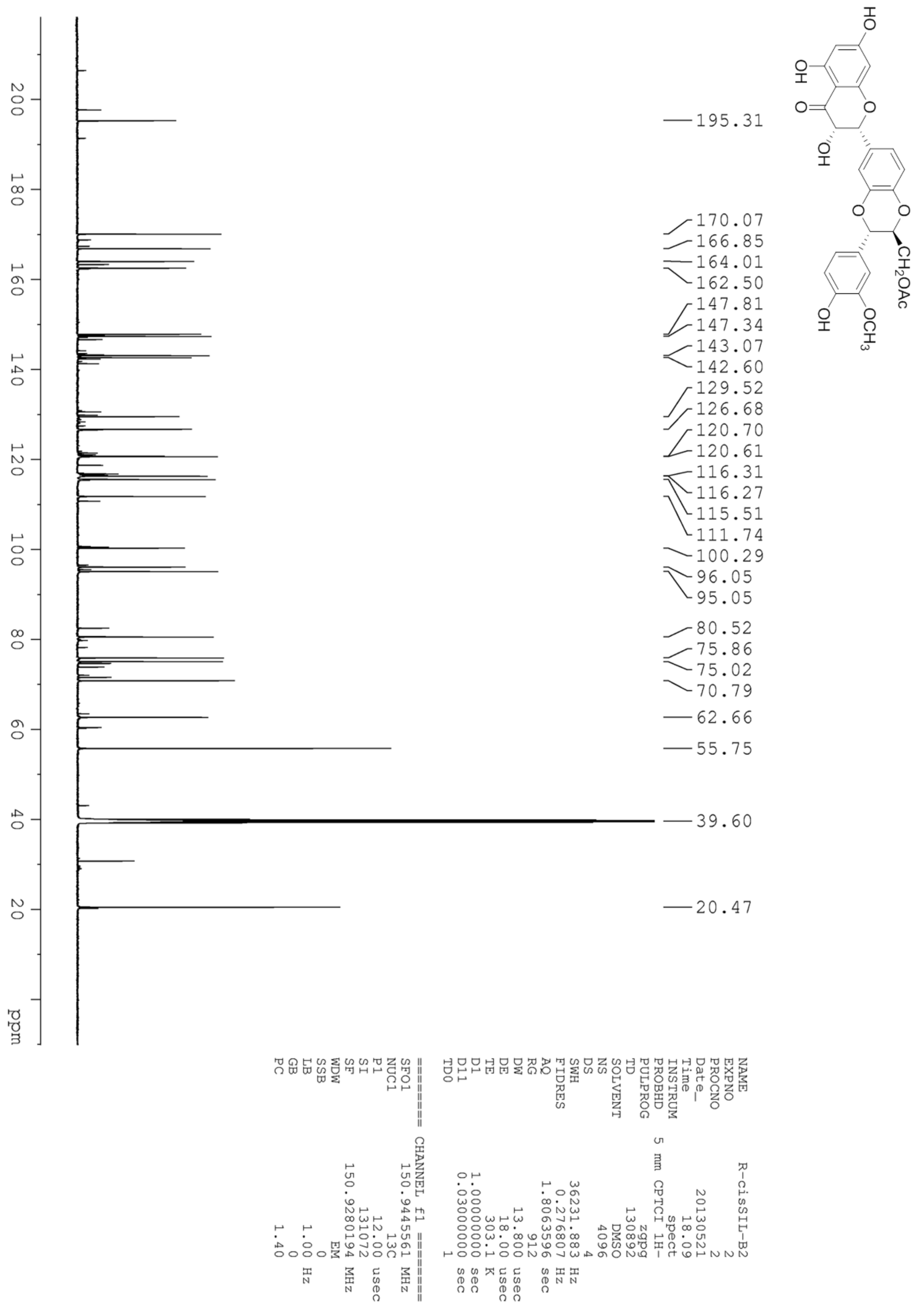

Figure S16. ^{13}C NMR Spectrum of **11**.

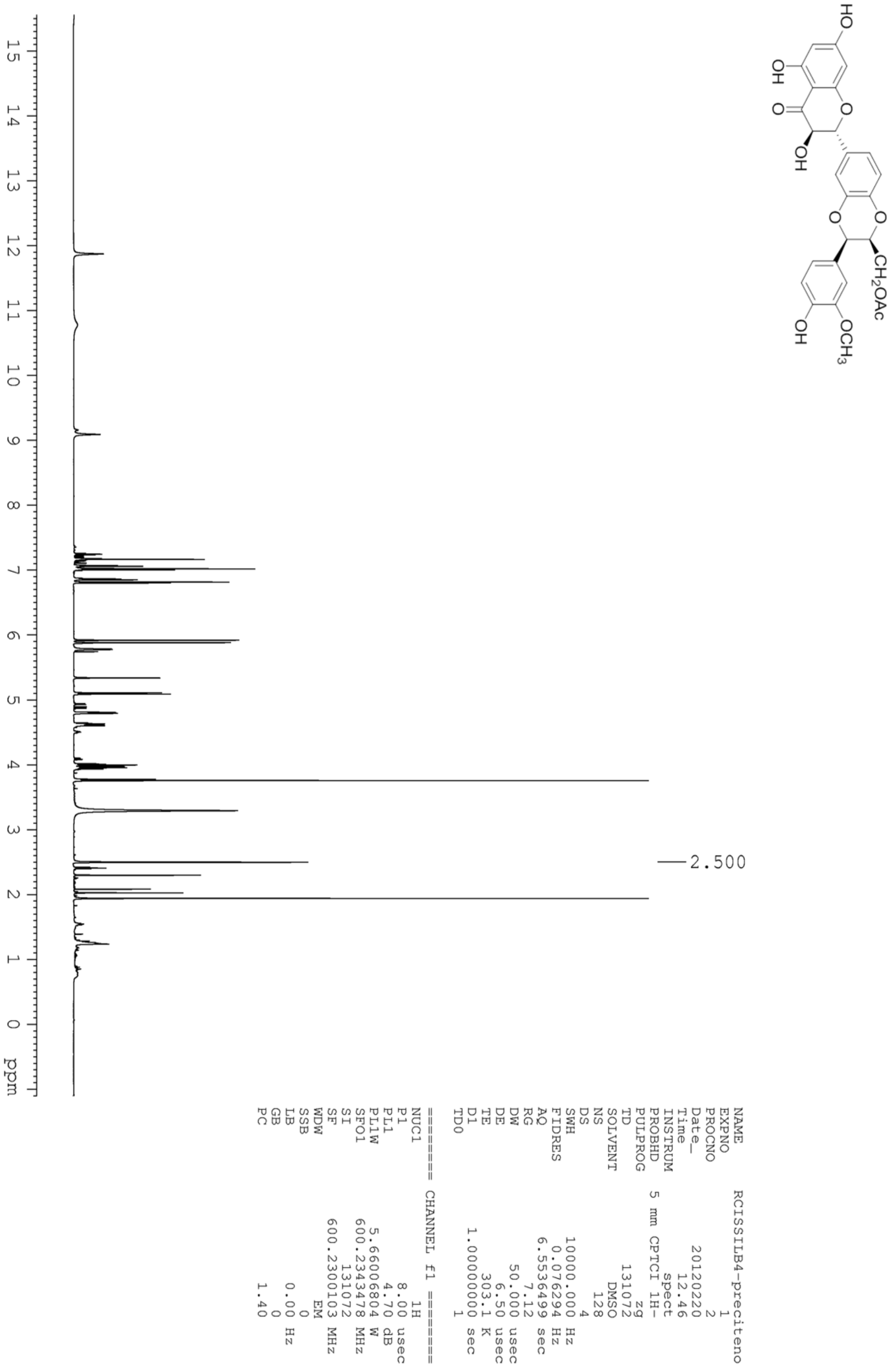

Figure S17. ^{1}H NMR Spectrum of **8**.

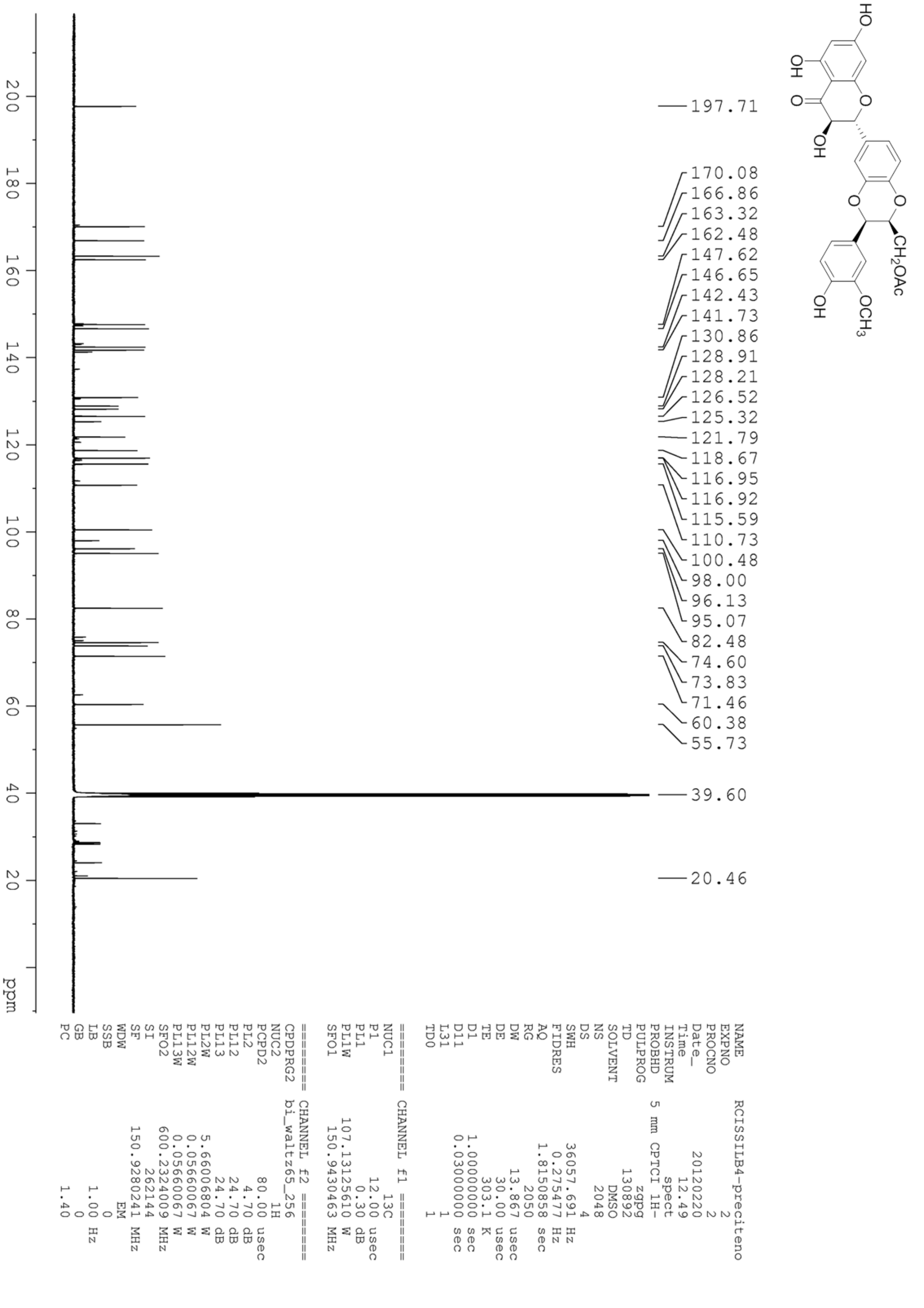

Figure S18. ^{13}C NMR Spectrum of **8**.

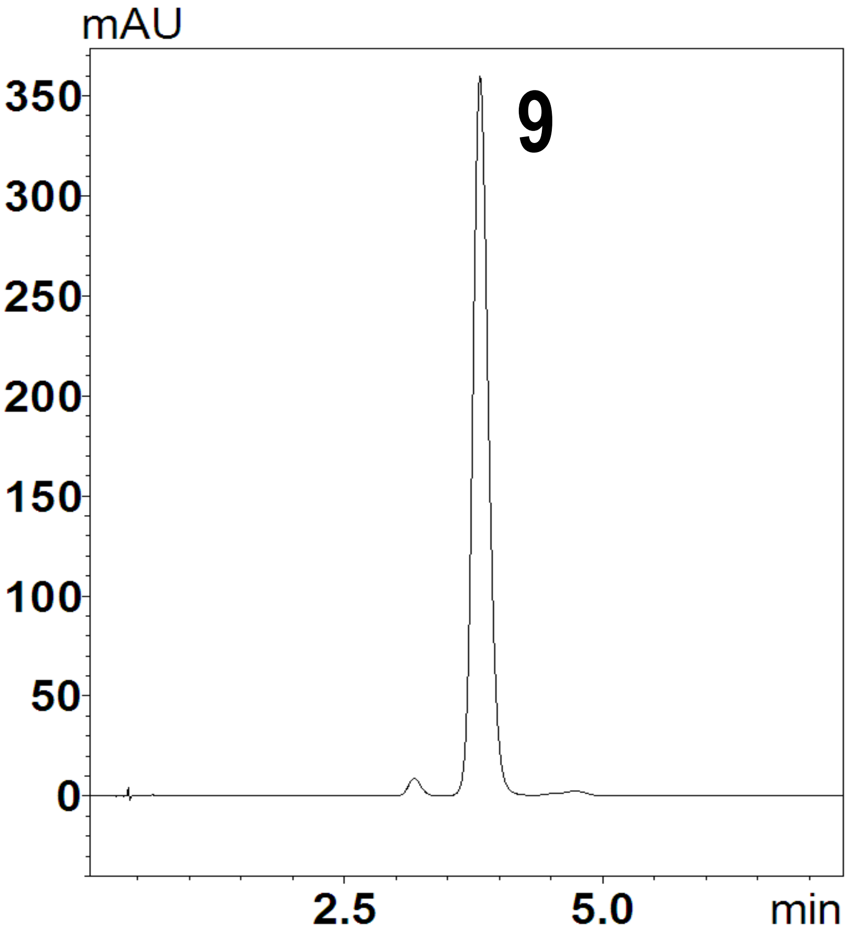

Figure S19. HPLC chromatogram of **9**.

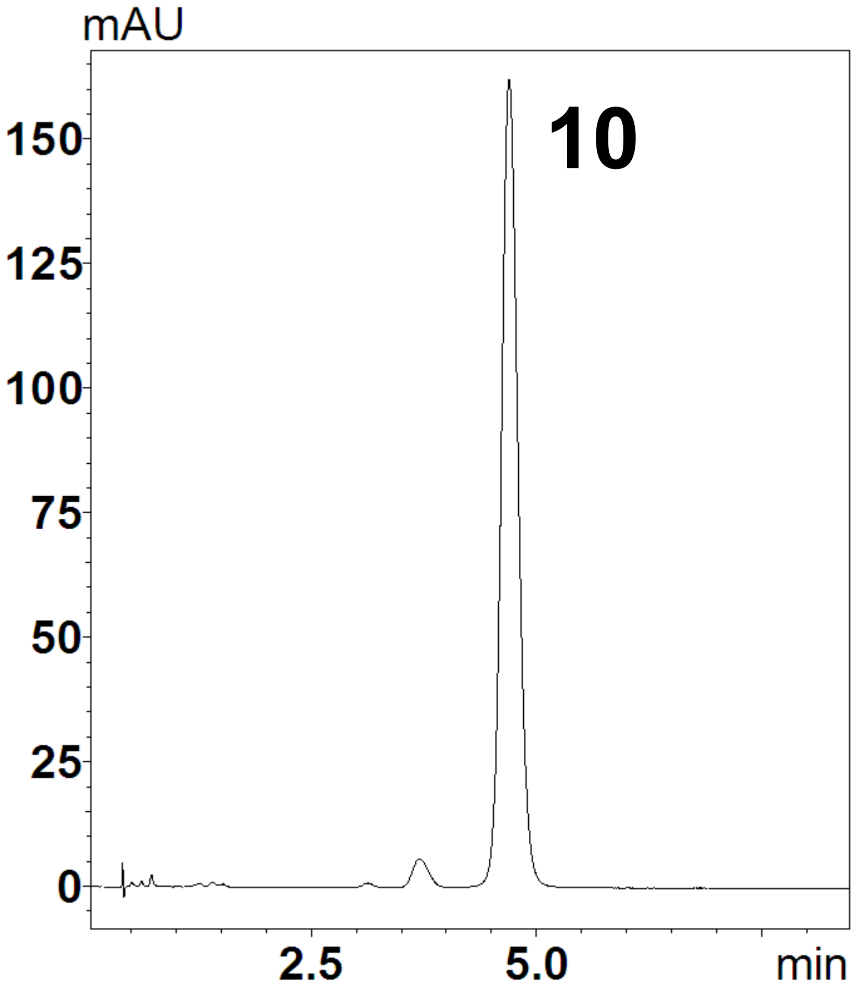

Figure S20. HPLC chromatogram of **10**.

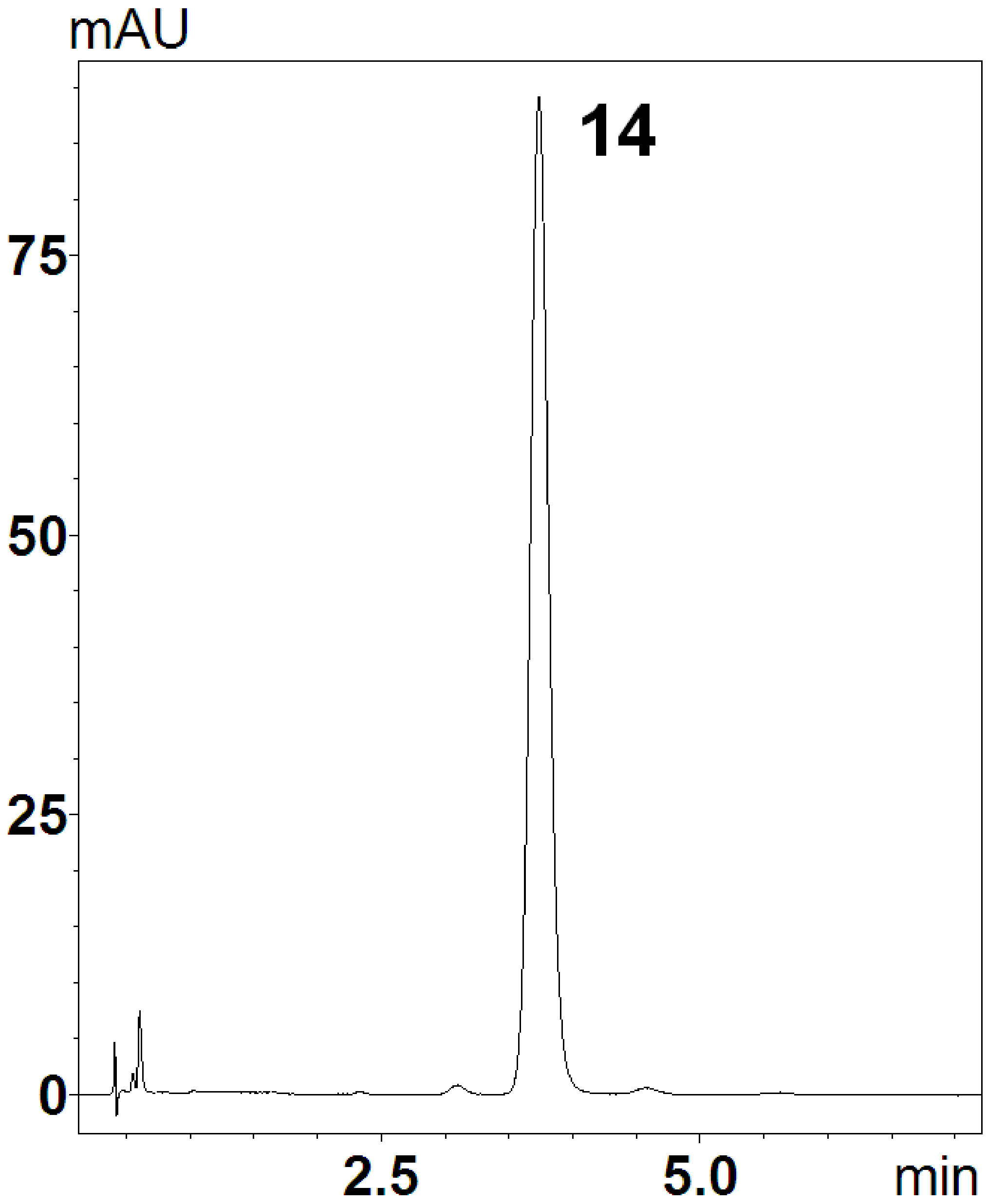

Figure S21. HPLC chromatogram of **14**.

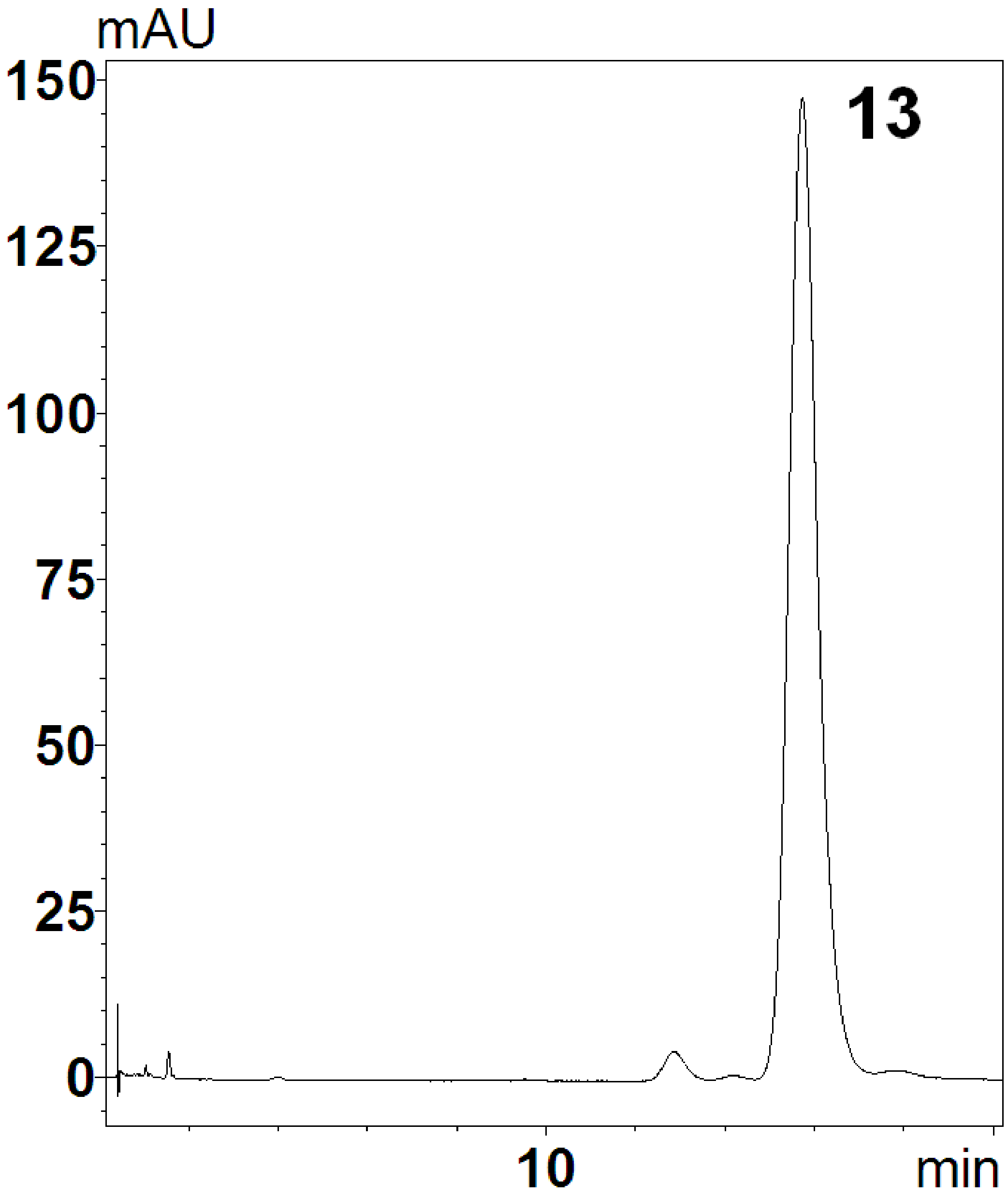

Figure S22. HPLC chromatogram of **13**.

Table S1. Retention Time and Purity of Compounds 9, 10, 13, and 14

Compound	Retention time [min]	HPLC purity [%]
**9**	3.8	97
**10**	4.7	96
**13**	15.7	96
**14**	3.7	98

Table S2. XYZ coordinates of optimized silybin A (**1a**) and B (**1b**) in (a) EtOAc and (b) DMF.

(a)

57

Coordinates of **1a** in EtOAc

C	-4.975485	-1.387645	-1.722550
C	-4.524320	-0.623602	-0.662845
C	-5.304198	-0.467882	0.510351
C	-6.585635	-1.081311	0.569397
C	-7.047877	-1.848732	-0.491024
C	-6.233029	-1.998204	-1.618182
O	-3.308353	-0.041156	-0.776065
C	-3.037447	1.096961	0.073970
C	-3.335470	0.706368	1.523604
C	-4.774556	0.243221	1.627910
C	-1.617650	1.520384	-0.162844
C	-0.574406	0.603037	-0.026241
C	0.742950	0.998504	-0.229697
C	1.025424	2.325431	-0.585752
C	-0.011088	3.245760	-0.706010
C	-1.326882	2.844689	-0.497911
O	1.731044	0.067197	-0.087554
C	3.056982	0.603504	0.047938
C	3.257656	1.686846	-1.011357
O	2.306269	2.739905	-0.806184
C	4.036482	-0.528537	-0.072203

C	5.043421	-0.675292	0.891793
C	5.984705	-1.689627	0.765568
C	5.922422	-2.577852	-0.323819
C	4.918717	-2.437395	-1.272178
C	3.980303	-1.412853	-1.149011
O	6.839108	-3.571853	-0.439651
O	7.009535	-1.942279	1.626473
C	7.160029	-1.096557	2.758604
C	4.618917	2.356106	-0.961177
O	4.727170	3.380845	-1.929927
O	-3.099160	1.783406	2.386963
O	-5.410705	0.484103	2.673848
O	-7.346593	-0.935361	1.659355
O	-6.627149	-2.729813	-2.680496
H	-4.382568	-1.498936	-2.621821
H	-8.024199	-2.318072	-0.432038
H	-7.507410	-3.099893	-2.529937
H	-6.842116	-0.364699	2.295285
H	-2.699806	-0.158044	1.786110
H	0.228505	4.268525	-0.978254
H	-2.130467	3.567272	-0.601666
H	-0.764883	-0.433554	0.234640
H	3.089932	1.253693	-2.007403
H	4.794768	2.749783	0.050559
H	5.395556	1.622670	-1.185521

H	4.005041	4.004909	-1.776701
H	5.080840	0.004097	1.736802
H	4.880488	-3.134869	-2.102508
H	3.193233	-1.317765	-1.890087
H	8.029484	-1.469608	3.297987
H	7.335619	-0.060229	2.450940
H	6.277602	-1.147885	3.405157
H	7.447454	-3.506754	0.313196
H	3.142711	1.071123	1.038622
H	-3.716651	1.910458	-0.214103
H	-3.802259	1.745908	3.058637

57

Coordinates of **1b** in EtOAc

C	-5.312467	1.451506	-0.961693
C	-4.528630	0.488990	-0.353548
C	-5.044467	-0.799689	-0.067623
C	-6.402363	-1.080837	-0.381481
C	-7.197410	-0.119861	-0.991116
C	-6.639445	1.130065	-1.279746
O	-3.249708	0.812853	-0.053020
C	-2.587689	0.027432	0.964700
C	-2.720403	-1.454270	0.603197
C	-4.188236	-1.805794	0.470816
C	-1.167518	0.501324	1.060991

C	-0.368431	0.571699	-0.081582
C	0.954780	0.986172	0.010858
C	1.491149	1.350789	1.254285
C	0.698368	1.271453	2.395117
C	-0.623705	0.849199	2.299435
O	1.695666	1.041787	-1.138444
C	3.105628	1.194026	-0.962518
C	3.370782	2.214204	0.152292
O	2.785184	1.764785	1.374354
O	-2.104350	-2.257967	1.570155
O	-4.568668	-2.943433	0.813482
O	-6.916730	-2.284213	-0.106215
O	-7.360578	2.106435	-1.868320
C	2.879270	3.611193	-0.221776
C	3.805649	-0.132685	-0.741031
C	5.208704	-0.161846	-0.794320
C	5.890184	-1.356659	-0.609050
C	5.178319	-2.547237	-0.368006
C	3.794486	-2.518994	-0.324680
C	3.109576	-1.314979	-0.512691
O	5.851169	-3.714409	-0.189531
O	7.242636	-1.521407	-0.642951
C	8.054322	-0.380730	-0.881048
O	3.143792	4.574754	0.778042
H	-4.920139	2.438846	-1.171204

H	-8.229652	-0.350011	-1.232782
H	-8.264602	1.808560	-2.037122
H	-6.197759	-2.821066	0.317282
H	-2.262257	-1.608989	-0.390079
H	1.130818	1.556847	3.348475
H	-1.235121	0.795323	3.194877
H	-0.758239	0.309151	-1.060465
H	-3.100096	0.200659	1.920655
H	-2.676371	-3.038413	1.672760
H	3.453676	1.624786	-1.907956
H	1.794855	3.607080	-0.358379
H	3.335492	3.892378	-1.181372
H	4.445197	2.250597	0.355502
H	5.763746	0.749064	-0.994734
H	3.254992	-3.443050	-0.144258
H	2.026417	-1.315414	-0.485705
H	9.083577	-0.736773	-0.869988
H	7.830387	0.061761	-1.857674
H	7.917192	0.368082	-0.093522
H	6.799749	-3.525103	-0.260096
H	4.098131	4.695393	0.856680

(b)

57

Coordinates of **1a** in DMF

C	-5.000954	-1.299026	-1.769733
C	-4.536055	-0.587006	-0.680335
C	-5.303016	-0.484208	0.507038
C	-6.585708	-1.096279	0.549696
C	-7.062371	-1.811824	-0.539853
C	-6.259382	-1.911251	-1.681366
O	-3.318691	-0.003837	-0.778834
C	-3.034800	1.092325	0.122076
C	-3.319326	0.632946	1.553682
C	-4.759481	0.172136	1.650891
C	-1.616209	1.521872	-0.111596
C	-0.569626	0.605763	0.013068
C	0.745785	1.004980	-0.196344
C	1.023555	2.335107	-0.545551
C	-0.015688	3.254264	-0.652929
C	-1.329836	2.849149	-0.439729
O	1.736397	0.074825	-0.067565
C	3.065002	0.611302	0.059269
C	3.253997	1.704173	-0.992204
O	2.301290	2.753320	-0.771372
C	4.042083	-0.520519	-0.082750
C	5.048747	-0.687208	0.878450

C	5.987078	-1.702213	0.734078
C	5.921065	-2.570819	-0.371051
C	4.917896	-2.410817	-1.316939
C	3.982701	-1.385248	-1.175608
O	6.835207	-3.565936	-0.504695
O	7.011100	-1.973077	1.588525
C	7.169884	-1.144097	2.733841
C	4.614513	2.375196	-0.946458
O	4.717352	3.401474	-1.916111
O	-3.067581	1.666584	2.466132
O	-5.384187	0.366726	2.713588
O	-7.334901	-0.998847	1.654318
O	-6.664592	-2.591621	-2.772316
H	-4.417721	-1.369781	-2.679524
H	-8.039350	-2.281033	-0.493172
H	-7.542736	-2.971598	-2.631783
H	-6.821528	-0.459218	2.309933
H	-2.686822	-0.246133	1.769045
H	0.219842	4.279336	-0.920093
H	-2.135241	3.570704	-0.536997
H	-0.755837	-0.432702	0.270064
H	3.079565	1.279448	-1.990641
H	4.794939	2.768582	0.063823
H	5.390767	1.642824	-1.175264
H	4.012206	4.039766	-1.743159

H	5.089886	-0.022128	1.734400
H	4.877092	-3.092237	-2.160518
H	3.197938	-1.273634	-1.916931
H	8.040692	-1.528525	3.262634
H	7.346818	-0.104228	2.440374
H	6.290273	-1.203054	3.383051
H	7.446090	-3.514964	0.247649
H	3.160338	1.068361	1.053469
H	-3.713897	1.920215	-0.120767
H	-3.763586	1.600924	3.143193

57

Coordinates of **1b** in DMF

C	-5.337529	1.466667	-0.901827
C	-4.540950	0.494391	-0.327187
C	-5.045801	-0.803927	-0.066696
C	-6.406075	-1.084368	-0.370923
C	-7.214409	-0.114412	-0.947207
C	-6.666924	1.145616	-1.212136
O	-3.259614	0.818466	-0.034383
C	-2.581100	0.010658	0.955276
C	-2.710026	-1.461338	0.555773
C	-4.177507	-1.817790	0.435716
C	-1.162621	0.491088	1.047460
C	-0.364385	0.559522	-0.096372

C	0.956812	0.981897	-0.008028
C	1.492556	1.355465	1.233593
C	0.700783	1.278342	2.375666
C	-0.619694	0.848909	2.283721
O	1.696273	1.035374	-1.157307
C	3.107366	1.192055	-0.982839
C	3.371551	2.219473	0.124402
O	2.784749	1.774986	1.350846
O	-2.074937	-2.287858	1.492389
O	-4.546945	-2.965825	0.756233
O	-6.910117	-2.298057	-0.118213
O	-7.399423	2.131658	-1.767776
C	2.877410	3.613630	-0.254311
C	3.807387	-0.133133	-0.750038
C	5.211524	-0.157940	-0.776615
C	5.892754	-1.351253	-0.579330
C	5.179073	-2.544219	-0.353472
C	3.794549	-2.520733	-0.337765
C	3.109848	-1.317877	-0.537594
O	5.852445	-3.709873	-0.162119
O	7.244594	-1.512259	-0.586116
C	8.059663	-0.368127	-0.807895
O	3.151337	4.580652	0.741628
H	-4.953396	2.461244	-1.092031
H	-8.248349	-0.343235	-1.182458

H	-8.304549	1.834395	-1.934197
H	-6.182539	-2.840264	0.283117
H	-2.266399	-1.588476	-0.447504
H	1.131591	1.570498	3.327888
H	-1.230258	0.798615	3.180006
H	-0.752674	0.286656	-1.073122
H	-3.083228	0.156714	1.920888
H	-2.642160	-3.073388	1.583809
H	3.454143	1.616060	-1.931098
H	1.791891	3.609264	-0.383388
H	3.328170	3.891254	-1.216442
H	4.444911	2.259410	0.328355
H	5.767719	0.754432	-0.966048
H	3.253488	-3.446565	-0.170747
H	2.026257	-1.321934	-0.533754
H	9.088779	-0.722439	-0.772959
H	7.855755	0.071721	-1.789624
H	7.901940	0.380787	-0.024902
H	6.802075	-3.518298	-0.216198
H	4.104399	4.729857	0.780717

Table S3. XYZ coordinates of different [BF_3-silybin A] complexes

61

Coordinates of Silybin A_O1-BF_3_EtOAc.xyz performed

C	-4.222464	-2.396500	1.853083
C	-3.859958	-1.451381	0.923677
C	-4.721694	-0.347743	0.601966
C	-5.990427	-0.265132	1.284389
C	-6.357545	-1.232913	2.200886
C	-5.472705	-2.280294	2.482851
O	-2.641928	-1.557117	0.370031
C	-2.455881	-0.958928	-0.928064
C	-2.900538	0.507370	-0.927415
C	-4.250327	0.653465	-0.248886
C	-1.018126	-1.141198	-1.316233
C	0.000965	-0.679799	-0.481374
C	1.333911	-0.838257	-0.843921
C	1.656581	-1.473770	-2.052258
C	0.641533	-1.921202	-2.892318
C	-0.690241	-1.758053	-2.525283
O	2.297899	-0.375747	0.003555
C	3.604031	-0.233123	-0.581062
C	3.921371	-1.493859	-1.384158
O	2.953884	-1.652100	-2.431247
C	4.585123	0.029830	0.524524
C	5.424850	1.149673	0.451304

C	6.363556	1.381751	1.449391
C	6.467029	0.497665	2.539003
C	5.628220	-0.605715	2.614996
C	4.691738	-0.840905	1.608834
O	7.380727	0.730594	3.514741
O	7.235321	2.426807	1.501484
C	7.199572	3.384743	0.452169
C	5.270030	-1.458015	-2.078998
O	5.487971	-2.625535	-2.846965
O	-2.952761	0.864414	-2.282462
O	-4.969973	1.709444	-0.451221
O	-6.840732	0.730984	1.055199
O	-5.773073	-3.235535	3.368801
H	-3.559945	-3.218032	2.093943
H	-7.318887	-1.155430	2.696723
H	-6.647487	-3.090762	3.757411
H	-6.429526	1.370958	0.435720
H	-2.185217	1.125809	-0.361148
H	0.911954	-2.406599	-3.824342
H	-1.475738	-2.114872	-3.183971
H	-0.217520	-0.194205	0.465009
H	3.864597	-2.369708	-0.723082
H	5.334202	-0.556254	-2.705089
H	6.064129	-1.416297	-1.331201
H	4.757606	-2.702382	-3.475430

H	5.333985	1.834109	-0.385467
H	5.715716	-1.273012	3.466103
H	4.033692	-1.700714	1.684007
H	7.967534	4.119250	0.690414
H	7.426045	2.916642	-0.511616
H	6.222504	3.877037	0.406336
H	7.859743	1.543230	3.288089
H	3.581304	0.619513	-1.273558
H	-3.100992	-1.489777	-1.639578
H	-3.230902	1.792130	-2.381047
B	-4.512829	3.160934	-0.795216
F	-3.383596	3.417288	-0.042674
F	-4.230004	3.222991	-2.163377
F	-5.592285	3.936251	-0.459516

61

Coordinates of Silybin A_O4-BF_3_EtOAc.xyz performed

C	-4.222464	-2.396500	1.853083
C	-3.859958	-1.451381	0.923677
C	-4.721694	-0.347743	0.601966
C	-5.990427	-0.265132	1.284389
C	-6.357545	-1.232913	2.200886
C	-5.472705	-2.280294	2.482851
O	-2.641928	-1.557117	0.370031
C	-2.455881	-0.958928	-0.928064

C	-2.900538	0.507370	-0.927415
C	-4.250327	0.653465	-0.248886
C	-1.018126	-1.141198	-1.316233
C	0.000965	-0.679799	-0.481374
C	1.333911	-0.838257	-0.843921
C	1.656581	-1.473770	-2.052258
C	0.641533	-1.921202	-2.892318
C	-0.690241	-1.758053	-2.525283
O	2.297899	-0.375747	0.003555
C	3.604031	-0.233123	-0.581062
C	3.921371	-1.493859	-1.384158
O	2.953884	-1.652100	-2.431247
C	4.585123	0.029830	0.524524
C	5.424850	1.149673	0.451304
C	6.363556	1.381751	1.449391
C	6.467029	0.497665	2.539003
C	5.628220	-0.605715	2.614996
C	4.691738	-0.840905	1.608834
O	7.380727	0.730594	3.514741
O	7.235321	2.426807	1.501484
C	7.199572	3.384743	0.452169
C	5.270030	-1.458015	-2.078998
O	5.487971	-2.625535	-2.846965
O	-2.952761	0.864414	-2.282462
O	-4.969973	1.709444	-0.451221

O	-6.840732	0.730984	1.055199
O	-5.773073	-3.235535	3.368801
H	-3.559945	-3.218032	2.093943
H	-7.318887	-1.155430	2.696723
H	-6.647487	-3.090762	3.757411
H	-6.429526	1.370958	0.435720
H	-2.185217	1.125809	-0.361148
H	0.911954	-2.406599	-3.824342
H	-1.475738	-2.114872	-3.183971
H	-0.217520	-0.194205	0.465009
H	3.864597	-2.369708	-0.723082
H	5.334202	-0.556254	-2.705089
H	6.064129	-1.416297	-1.331201
H	4.757606	-2.702382	-3.475430
H	5.333985	1.834109	-0.385467
H	5.715716	-1.273012	3.466103
H	4.033692	-1.700714	1.684007
H	7.967534	4.119250	0.690414
H	7.426045	2.916642	-0.511616
H	6.222504	3.877037	0.406336
H	7.859743	1.543230	3.288089
H	3.581304	0.619513	-1.273558
H	-3.100992	-1.489777	-1.639578
H	-3.230902	1.792130	-2.381047
B	-4.512829	3.160934	-0.795216

F	-3.383596	3.417288	-0.042674
F	-4.230004	3.222991	-2.163377
F	-5.592285	3.936251	-0.459516

61

Coordinates of Silybin A_O9-BF_3_EtOAc.xyz performed

C	5.051021	2.050866	-1.311894
C	4.723415	0.967984	-0.519494
C	5.644988	0.441399	0.419096
C	6.937736	1.026028	0.513105
C	7.276362	2.115130	-0.278610
C	6.327081	2.616309	-1.174939
O	3.485956	0.433814	-0.660021
C	3.299144	-0.923678	-0.214533
C	3.796776	-1.045530	1.228430
C	5.249318	-0.617483	1.289274
C	1.841853	-1.258838	-0.370932
C	0.871243	-0.373098	0.088917
C	-0.482501	-0.687234	-0.028238
C	-0.836027	-1.905706	-0.608608
C	0.116169	-2.787399	-1.093808
C	1.461984	-2.465186	-0.966770
O	-1.384713	0.230923	0.395307
C	-2.786100	-0.060878	0.227762
C	-2.976247	-0.952346	-0.996592

O	-2.208124	-2.200535	-0.752578
C	-3.520436	1.246104	0.115223
C	-4.639245	1.476405	0.927706
C	-5.348555	2.665120	0.808102
C	-4.939949	3.640012	-0.121609
C	-3.825863	3.412496	-0.917860
C	-3.118775	2.216448	-0.801798
O	-5.630726	4.801476	-0.229417
O	-6.445644	3.019497	1.530912
C	-6.936628	2.104575	2.502226
C	-4.417362	-1.273932	-1.351613
O	-4.516763	-2.015526	-2.544829
O	3.643964	-2.355639	1.695429
O	6.009685	-1.185481	2.098018
O	7.830236	0.538628	1.380833
O	6.597669	3.667881	-1.974137
H	4.349435	2.445335	-2.036289
H	8.262519	2.558762	-0.192031
H	7.501416	3.979979	-1.831211
H	7.400886	-0.224018	1.847739
H	3.225324	-0.332707	1.849425
H	-0.199533	-3.713148	-1.559490
H	2.214905	-3.151667	-1.338990
H	1.140845	0.581355	0.528802
H	-2.490030	-0.514575	-1.874183

H	-4.931533	-1.765490	-0.518683
H	-4.909629	-0.313979	-1.524547
H	-4.318185	-2.938282	-2.331321
H	-4.943396	0.727272	1.651050
H	-3.519598	4.178421	-1.622591
H	-2.240023	2.054098	-1.417617
H	-7.805617	2.582731	2.951761
H	-7.237586	1.162142	2.032807
H	-6.183027	1.912984	3.273153
H	-6.362971	4.774908	0.406620
H	-3.130308	-0.609364	1.110471
H	3.901955	-1.585895	-0.850182
H	4.428649	-2.539522	2.240740
B	-2.873080	-3.485043	0.101652
F	-3.510597	-2.916643	1.168673
F	-3.729463	-4.052131	-0.813495
F	-1.810811	-4.263432	0.423145

61

Coordinates of Silybin A_O12-BF_3_EtOAc.xyz performed

C	4.468282	2.200628	-1.273027
C	4.327479	0.984493	-0.633376
C	5.373405	0.446810	0.156726
C	6.596941	1.164530	0.254575
C	6.748404	2.387517	-0.384998

C	5.679773	2.892870	-1.132277
O	3.150520	0.328450	-0.776464
C	3.149753	-1.088541	-0.525568
C	3.773429	-1.353663	0.849105
C	5.168651	-0.763596	0.883246
C	1.732620	-1.577576	-0.626965
C	0.689954	-0.822512	-0.086099
C	-0.598617	-1.328624	-0.125936
C	-0.877828	-2.552267	-0.729906
C	0.158825	-3.300556	-1.274190
C	1.460940	-2.815963	-1.211981
O	-1.639539	-0.615516	0.489879
C	-3.059500	-0.916208	0.108452
C	-3.059163	-1.888467	-1.061259
O	-2.169770	-2.982351	-0.807050
C	-3.778009	0.362865	-0.192104
C	-4.918961	0.692384	0.553790
C	-5.636151	1.841630	0.252027
C	-5.210806	2.684098	-0.793007
C	-4.073084	2.363385	-1.521530
C	-3.361556	1.202386	-1.226115
O	-5.908996	3.808171	-1.079351
O	-6.755753	2.277420	0.890017
C	-7.244327	1.515284	1.986811
C	-4.434648	-2.497511	-1.286256

O	-4.434502	-3.358225	-2.403743
O	3.810017	-2.727329	1.116353
O	6.050391	-1.342995	1.548116
O	7.605281	0.670763	0.980270
O	5.765161	4.073392	-1.778064
H	3.670733	2.606054	-1.883024
H	7.684144	2.928916	-0.296371
H	6.631926	4.477630	-1.636880
H	7.301374	-0.196368	1.354354
H	3.174998	-0.816967	1.606393
H	-0.070669	-4.248475	-1.748668
H	2.269837	-3.403166	-1.634521
H	0.879064	0.141095	0.372215
H	-2.730168	-1.385909	-1.980455
H	-4.749365	-3.025371	-0.375109
H	-5.155193	-1.704114	-1.493656
H	-3.806167	-4.071723	-2.230422
H	-5.228489	0.051527	1.371710
H	-3.753269	3.028921	-2.316191
H	-2.469248	0.971584	-1.799593
H	-8.124924	2.043371	2.349376
H	-7.527184	0.507309	1.665577
H	-6.495869	1.456797	2.783636
H	-6.654839	3.864612	-0.461011
H	-3.480134	-1.405949	0.988108

H	3.767704	-1.580805	-1.288872
H	4.649133	-2.886877	1.582518
B	-1.356840	-0.014602	2.056928
F	-2.606634	0.066840	2.593878
F	-0.749288	1.185721	1.835945
F	-0.559245	-0.956306	2.635258

61

Coordinates of Silybin A_O1-BF_3_DMF.xyz performed

C	4.804334	-0.714756	1.684944
C	4.305502	-0.570198	0.404738
C	5.024232	-1.052943	-0.717505
C	6.295059	-1.656495	-0.510227
C	6.805921	-1.803196	0.771912
C	6.048715	-1.339451	1.852664
O	3.101243	0.035389	0.257580
C	2.794312	0.588781	-1.044388
C	3.013934	-0.496257	-2.101489
C	4.443891	-0.986945	-2.018698
C	1.389373	1.113232	-1.005348
C	0.335257	0.283115	-0.617566
C	-0.971286	0.758723	-0.613996
C	-1.232316	2.083797	-0.993913
C	-0.185556	2.907949	-1.396265
C	1.119889	2.426418	-1.398828

O	-1.969915	-0.085685	-0.223681
C	-3.298408	0.333385	-0.582891
C	-3.453211	1.814784	-0.240273
O	-2.500755	2.582756	-0.988945
C	-4.276696	-0.547448	0.139632
C	-5.296203	-1.184291	-0.581554
C	-6.230189	-1.970798	0.082514
C	-6.147765	-2.136242	1.477549
C	-5.133024	-1.511378	2.189203
C	-4.201532	-0.715353	1.522297
O	-7.058134	-2.911431	2.121022
O	-7.265441	-2.642224	-0.491786
C	-7.439510	-2.534543	-1.899596
C	-4.811286	2.390349	-0.597232
O	-4.882230	3.772526	-0.299679
O	2.731398	-0.003929	-3.382590
O	5.028104	-1.323980	-3.068547
O	6.999362	-2.104445	-1.556032
O	6.486656	-1.452859	3.122458
H	4.258186	-0.342679	2.542232
H	7.773467	-2.271492	0.917952
H	7.352214	-1.883335	3.148685
H	6.467705	-1.912425	-2.371663
H	2.364971	-1.355419	-1.856295
H	-0.408076	3.929076	-1.688453

H	1.931704	3.078630	-1.705917
H	0.509635	-0.743934	-0.311089
H	-3.253965	1.961456	0.830324
H	-5.020136	2.204993	-1.660364
H	-5.583101	1.897687	-0.003216
H	-4.181351	4.215932	-0.796378
H	-5.350417	-1.061902	-1.658089
H	-5.079765	-1.653770	3.263678
H	-3.406276	-0.238239	2.086028
H	-8.316102	-3.134322	-2.139502
H	-7.614190	-1.493891	-2.191361
H	-6.567437	-2.928838	-2.431253
H	-7.678223	-3.256941	1.459225
H	-3.416444	0.215730	-1.668383
H	3.491343	1.414459	-1.238898
H	3.403870	-0.388910	-3.971478
B	1.899026	1.993127	2.636761
F	2.170305	0.920488	3.356275
F	0.645050	2.321888	2.398386
F	2.878463	2.754527	2.190664

61

Coordinates of Silybin A_O4-BF_3_DMF.xyz performed

C	4.116531	-3.062664	-0.486060
C	3.796222	-1.769450	-0.146334

C	4.681739	-0.676839	-0.431723
C	5.939691	-0.985914	-1.065931
C	6.261585	-2.289769	-1.393014
C	5.347673	-3.312373	-1.112959
O	2.600074	-1.561324	0.427998
C	2.430824	-0.359226	1.205133
C	2.862090	0.861608	0.387698
C	4.252084	0.632385	-0.182236
C	0.998024	-0.301717	1.647976
C	-0.032380	-0.308464	0.705906
C	-1.360501	-0.255275	1.114118
C	-1.667467	-0.206841	2.482307
C	-0.641429	-0.186735	3.422299
C	0.685813	-0.235934	3.007669
O	-2.334952	-0.267154	0.159631
C	-3.639351	0.142447	0.609324
C	-3.939289	-0.549396	1.938575
O	-2.959333	-0.165956	2.913589
C	-4.628352	-0.192703	-0.469714
C	-5.493536	0.800973	-0.948711
C	-6.436248	0.491018	-1.921873
C	-6.517223	-0.817427	-2.433458
C	-5.654279	-1.798189	-1.964205
C	-4.714050	-1.487359	-0.981876
O	-7.435166	-1.113633	-3.389023

O	-7.332056	1.354419	-2.473834
C	-7.332264	2.704376	-2.024800
C	-5.280708	-0.171052	2.539263
O	-5.489598	-0.811948	3.784020
O	2.804139	1.956419	1.256169
O	5.031662	1.615743	-0.475324
O	6.823062	-0.035277	-1.364716
O	5.603215	-4.588968	-1.417961
H	3.436620	-3.875927	-0.266145
H	7.212490	-2.497168	-1.870738
H	6.468385	-4.682521	-1.842249
H	6.453676	0.836454	-1.112986
H	2.186225	0.981532	-0.474352
H	-0.899328	-0.146151	4.475535
H	1.479373	-0.228013	3.748342
H	0.173128	-0.360832	-0.359129
H	-3.885214	-1.638334	1.801622
H	-5.340157	0.921431	2.643720
H	-6.082344	-0.498506	1.874794
H	-4.769490	-0.550303	4.373492
H	-5.421403	1.810750	-0.558978
H	-5.725478	-2.800800	-2.373213
H	-4.037240	-2.258934	-0.628976
H	-8.118636	3.206214	-2.586494
H	-7.553223	2.758519	-0.953878

H	-6.369950	3.183875	-2.231247
H	-7.935045	-0.305079	-3.584550
H	-3.621678	1.227070	0.780029
H	3.085578	-0.425747	2.083604
H	2.758577	2.765312	0.723648
B	4.841603	3.150604	-0.586123
F	3.498858	3.421902	-0.867467
F	5.240474	3.713362	0.606775
F	5.660394	3.506146	-1.637074

61

Coordinates of Silybin A_O9-BF_3_DMF.xyz performed

C	5.005590	2.088248	-1.287160
C	4.678019	0.992384	-0.512805
C	5.596423	0.456341	0.423617
C	6.885784	1.045703	0.532953
C	7.224996	2.147334	-0.240234
C	6.278462	2.657643	-1.134911
O	3.444003	0.454257	-0.668991
C	3.263248	-0.911643	-0.244200
C	3.750302	-1.048770	1.200556
C	5.200525	-0.616287	1.276185
C	1.810003	-1.255352	-0.418820
C	0.828689	-0.418480	0.108735
C	-0.519756	-0.744535	-0.027833

C	-0.856522	-1.922717	-0.695526
C	0.103693	-2.751360	-1.250703
C	1.446205	-2.417859	-1.104436
O	-1.438598	0.116531	0.471858
C	-2.837818	-0.133236	0.217686
C	-2.981275	-0.972221	-1.048424
O	-2.227994	-2.235769	-0.830570
C	-3.537258	1.195092	0.125140
C	-4.681932	1.420893	0.901848
C	-5.358490	2.630737	0.803004
C	-4.889969	3.630556	-0.070238
C	-3.750222	3.407192	-0.830964
C	-3.075705	2.190497	-0.735642
O	-5.549025	4.812956	-0.158563
O	-6.474368	2.982764	1.496008
C	-7.025252	2.041701	2.410499
C	-4.401137	-1.236119	-1.487988
O	-4.346183	-1.991885	-2.682192
O	3.599033	-2.365951	1.651814
O	5.958786	-1.193009	2.081445
O	7.775688	0.548452	1.399451
O	6.548224	3.721449	-1.917081
H	4.306371	2.489803	-2.010120
H	8.208379	2.594598	-0.142785
H	7.449778	4.037375	-1.766057

H	7.345775	-0.222400	1.852317
H	3.173210	-0.346423	1.827611
H	-0.203497	-3.643050	-1.782986
H	2.208224	-3.060515	-1.532499
H	1.087278	0.502859	0.620235
H	-2.446670	-0.502928	-1.880745
H	-4.976925	-1.744606	-0.710655
H	-4.848247	-0.249354	-1.660930
H	-5.246584	-2.227780	-2.935056
H	-5.033286	0.650679	1.580000
H	-3.397705	4.191544	-1.492671
H	-2.178760	2.031305	-1.325653
H	-7.900007	2.523526	2.844339
H	-7.328305	1.126644	1.891613
H	-6.307280	1.801704	3.201257
H	-6.307198	4.780863	0.446602
H	-3.234746	-0.703007	1.064328
H	3.875451	-1.559339	-0.885165
H	4.382378	-2.554241	2.197775
B	-2.731621	-3.465315	0.212506
F	-4.047606	-3.662590	-0.074466
F	-1.922319	-4.505993	-0.134503
F	-2.499592	-2.969585	1.467775

61

Coordinates of Silybin A_O12-BF_3_DMF.xyz performed

C	4.593173	2.073351	-1.429408
C	4.378431	0.930430	-0.684041
C	5.358418	0.450496	0.219949
C	6.592589	1.148537	0.325493
C	6.818337	2.298239	-0.418707
C	5.813427	2.749120	-1.280997
O	3.193607	0.289610	-0.836101
C	3.144924	-1.100454	-0.452146
C	3.668945	-1.235744	0.979692
C	5.075929	-0.677955	1.045319
C	1.729615	-1.574661	-0.618375
C	0.678994	-0.867833	-0.027255
C	-0.612508	-1.347306	-0.161357
C	-0.889389	-2.488599	-0.911844
C	0.154323	-3.191145	-1.500662
C	1.460336	-2.737942	-1.341745
O	-1.666857	-0.697145	0.502450
C	-3.077480	-0.910187	0.027691
C	-3.040857	-1.738257	-1.247280
O	-2.184211	-2.876568	-1.083045

C	-3.744181	0.418532	-0.151802
C	-4.949449	0.667847	0.520222
C	-5.618521	1.867423	0.318306
C	-5.077805	2.838641	-0.546587
C	-3.877198	2.594602	-1.200247
C	-3.214727	1.383366	-1.009658
O	-5.729152	4.011654	-0.733975
O	-6.791823	2.237306	0.896304
C	-7.422940	1.323585	1.786795
C	-4.414737	-2.282752	-1.607290
O	-4.374290	-2.996570	-2.824385
O	3.644170	-2.574262	1.391474
O	5.898214	-1.208594	1.818787
O	7.540042	0.707237	1.160783
O	5.972194	3.855306	-2.034269
H	3.847061	2.432425	-2.127441
H	7.760625	2.826859	-0.323043
H	6.842393	4.250284	-1.885219
H	7.189487	-0.112143	1.596409
H	3.044106	-0.607246	1.638393
H	-0.071403	-4.074904	-2.087392

H	2.275013	-3.286749	-1.803595
H	0.861825	0.036969	0.541034
H	-2.658380	-1.138787	-2.083856
H	-4.781468	-2.913219	-0.786007
H	-5.110698	-1.453162	-1.744862
H	-3.774426	-3.745897	-2.710852
H	-5.349818	-0.077139	1.198721
H	-3.471173	3.356274	-1.857435
H	-2.274514	1.209540	-1.523791
H	-8.335735	1.814463	2.120429
H	-7.673551	0.390242	1.272497
H	-6.781587	1.114536	2.648838
H	-6.538851	3.996482	-0.198631
H	-3.550041	-1.485525	0.824983
H	3.802781	-1.669558	-1.121965
H	4.450785	-2.707390	1.919483
B	-1.439914	-0.306233	2.122648
F	-2.706742	-0.236690	2.623543
F	-0.781001	0.890665	2.086675
F	-0.699359	-1.338084	2.624390

Table S3. XYZ coordinates of different [BF_3-silybin A] complexes

61

Coordinates of Silybin A_O1-BF_3_EtOAc.xyz performed

C	-4.649144	-1.649890	-1.519362
C	-4.351173	-0.650529	-0.623509
C	-5.091171	-0.484476	0.568627
C	-6.207631	-1.335585	0.798715
C	-6.525161	-2.345249	-0.100511
C	-5.732848	-2.500050	-1.236874
O	-3.309893	0.218305	-0.942140
C	-2.992216	1.361499	-0.049538
C	-3.274988	0.987775	1.408377
C	-4.663045	0.428372	1.575982
C	-1.551992	1.737098	-0.234404
C	-0.551775	0.769236	-0.140687
C	0.786924	1.131729	-0.237957
C	1.132761	2.477769	-0.426325
C	0.134993	3.445828	-0.497910
C	-1.202280	3.077650	-0.407261
O	1.733072	0.152199	-0.153853
C	3.065066	0.617964	0.120908
C	3.368063	1.806823	-0.790404
O	2.435844	2.864834	-0.527281
C	4.012943	-0.530647	-0.070693
C	4.913463	-0.860748	0.951440

C	5.822763	-1.895756	0.769448
C	5.836025	-2.618457	-0.437622
C	4.937999	-2.295767	-1.445599
C	4.030675	-1.252357	-1.264031
O	6.720961	-3.632904	-0.609076
O	6.745662	-2.320620	1.676650
C	6.798173	-1.668206	2.937922
C	4.742002	2.413057	-0.570467
O	4.949643	3.541845	-1.396755
O	-3.088821	2.122855	2.203811
O	-5.316114	0.718367	2.595307
O	-6.946724	-1.187449	1.900529
O	-5.969917	-3.461699	-2.147684
H	-4.093280	-1.772245	-2.437709
H	-7.372514	-2.993020	0.096876
H	-6.732768	-3.997213	-1.890476
H	-6.572105	-0.427287	2.414578
H	-2.578721	0.184204	1.704343
H	0.423153	4.481941	-0.641895
H	-1.974518	3.836772	-0.477768
H	-0.788932	-0.281562	-0.003489
H	3.257177	1.498303	-1.839098
H	4.859338	2.674497	0.491421
H	5.509554	1.682446	-0.832225
H	4.239190	4.171893	-1.215974

H	4.893673	-0.307177	1.884189
H	4.956228	-2.868324	-2.367098
H	3.324658	-1.015081	-2.053404
H	7.590317	-2.162266	3.498689
H	7.040281	-0.606815	2.818832
H	5.848124	-1.775320	3.472058
H	7.251165	-3.707263	0.199915
H	3.100885	0.964392	1.162963
H	-3.653041	2.180531	-0.349634
H	-3.807592	2.107935	2.860448
B	-3.144261	0.602468	-2.624900
H	-2.535768	-0.337079	-3.068125
H	-2.530422	1.630299	-2.594091
H	-4.293048	0.696730	-2.974483

61

Coordinates of Silybin A_O4-BF_3_EtOAc.xyz performed

C	-4.737220	-1.128305	-2.075602
C	-4.330771	-0.535220	-0.902063
C	-5.174231	-0.476669	0.267224
C	-6.542326	-0.903735	0.090996
C	-6.943286	-1.484609	-1.104263
C	-6.044201	-1.621824	-2.162112
O	-3.075127	-0.041737	-0.893817
C	-2.827297	1.054147	0.008791

C	-3.153842	0.546245	1.400243
C	-4.563747	-0.024297	1.460991
C	-1.406775	1.502386	-0.164171
C	-0.360699	0.579615	-0.113827
C	0.957897	1.003029	-0.240266
C	1.238163	2.364428	-0.429598
C	0.197100	3.286949	-0.467986
C	-1.119636	2.858021	-0.338545
O	1.950433	0.067349	-0.185887
C	3.263424	0.595728	0.065602
C	3.491396	1.796397	-0.852589
O	2.519417	2.810871	-0.565389
C	4.264325	-0.504351	-0.139217
C	5.205248	-0.774354	0.864000
C	6.166976	-1.758208	0.668874
C	6.192517	-2.490644	-0.532139
C	5.253933	-2.228837	-1.520649
C	4.294421	-1.235349	-1.326526
O	7.129071	-3.455157	-0.716700
O	7.133481	-2.120879	1.557172
C	7.178308	-1.453807	2.811247
C	4.841676	2.464489	-0.669114
O	4.974363	3.600995	-1.500465
O	-2.984538	1.580520	2.329040
O	-4.990533	-0.067734	2.656108

O	-7.514483	-0.697877	0.973475
O	-6.395989	-2.187659	-3.326219
H	-4.067283	-1.176596	-2.924700
H	-7.976620	-1.799513	-1.199491
H	-7.316635	-2.483958	-3.301454
H	-7.216819	-0.592606	1.916151
H	-2.484218	-0.305132	1.623003
H	0.434723	4.335644	-0.613542
H	-1.925786	3.584211	-0.375164
H	-0.548830	-0.481271	0.019968
H	3.368171	1.481364	-1.898162
H	4.975463	2.732151	0.389178
H	5.634264	1.769329	-0.951722
H	4.240473	4.197124	-1.299449
H	5.175633	-0.214661	1.792831
H	5.282607	-2.808557	-2.437397
H	3.558801	-1.044489	-2.101302
H	8.011732	-1.894905	3.356093
H	7.354379	-0.381214	2.677137
H	6.249712	-1.611367	3.370009
H	7.681320	-3.492497	0.080038
H	3.300231	0.946881	1.106091
H	-3.509525	1.876964	-0.242203
H	-3.446477	1.317511	3.139948
B	-6.009944	-0.987638	3.389226

H	-6.002780	-2.061855	2.829932
H	-5.640253	-1.024515	4.536567
H	-7.099436	-0.426729	3.347169

61

Coordinates of Silybin A_O9-BF_3_EtOAc.xyz performed

C	-5.095844	-0.983935	-1.951544
C	-4.603563	-0.481281	-0.762828
C	-5.334666	-0.607054	0.444544
C	-6.613032	-1.228968	0.410330
C	-7.116892	-1.736421	-0.779653
C	-6.348066	-1.614943	-1.941984
O	-3.392077	0.123595	-0.786476
C	-3.086981	1.032831	0.289858
C	-3.324318	0.315528	1.622201
C	-4.760308	-0.167191	1.673815
C	-1.671928	1.501508	0.104741
C	-0.654291	0.582203	-0.145290
C	0.658560	1.013686	-0.298930
C	0.952610	2.374753	-0.206031
C	-0.047322	3.296309	0.071449
C	-1.358635	2.857012	0.222003
O	1.637952	0.096383	-0.532328
C	2.901806	0.509426	0.004864
C	3.319515	1.834016	-0.622580

O	2.255659	2.833747	-0.423388
C	3.906899	-0.576944	-0.249510
C	4.642833	-1.100722	0.821802
C	5.580169	-2.101243	0.595776
C	5.781827	-2.596995	-0.705264
C	5.042829	-2.084045	-1.762547
C	4.110741	-1.072946	-1.536099
O	6.691864	-3.579460	-0.921077
O	6.361051	-2.696063	1.540174
C	6.224371	-2.272089	2.889436
C	4.595967	2.406418	-0.010729
O	5.655179	2.118673	-0.903208
O	-3.046758	1.163169	2.701003
O	-5.353175	-0.175522	2.771175
O	-7.330175	-1.344282	1.532831
O	-6.782200	-2.086423	-3.128090
H	-4.539407	-0.878755	-2.874520
H	-8.090393	-2.215150	-0.791355
H	-7.654605	-2.492051	-3.034645
H	-6.802189	-0.929509	2.262846
H	-2.680965	-0.582048	1.648227
H	0.200352	4.348302	0.137621
H	-2.140683	3.581092	0.425945
H	-0.859267	-0.479500	-0.235844
H	3.445001	1.724765	-1.703228

H	4.465732	3.485344	0.122433
H	4.777099	1.957356	0.975279
H	6.487727	2.366702	-0.484572
H	4.472739	-0.727872	1.826384
H	5.206594	-2.481621	-2.758639
H	3.534978	-0.683593	-2.369454
H	6.932917	-2.866670	3.464265
H	6.469461	-1.209502	2.991177
H	5.208772	-2.455970	3.255541
H	7.097487	-3.804567	-0.069051
H	2.779741	0.655782	1.087488
H	-3.771075	1.889806	0.227693
H	-3.730858	0.979561	3.368488
B	2.413822	4.238502	-1.359372
H	3.527140	4.141241	-1.803243
H	1.534881	4.123721	-2.176421
H	2.267436	5.136603	-0.567464

61

Coordinates of Silybin A_O12-BF_3_EtOAc.xyz performed

C	4.627405	1.990575	-1.347073
C	4.380501	0.881025	-0.561860
C	5.340200	0.416658	0.371579
C	6.587744	1.092125	0.466011
C	6.845226	2.207843	-0.319052

C	5.859094	2.646444	-1.208632
O	3.185507	0.259191	-0.705165
C	3.095412	-1.109704	-0.262360
C	3.605103	-1.199667	1.179287
C	5.023568	-0.671411	1.238006
C	1.668064	-1.552014	-0.411568
C	0.632701	-0.733265	0.041384
C	-0.680121	-1.169961	-0.051212
C	-0.979695	-2.411423	-0.614219
C	0.053229	-3.229283	-1.060803
C	1.371664	-2.802772	-0.955874
O	-1.698638	-0.349287	0.445973
C	-3.103382	-0.784341	0.267858
C	-3.179234	-1.737732	-0.915051
O	-2.270975	-2.827856	-0.736059
C	-3.963374	0.427785	0.071454
C	-5.097963	0.601330	0.876396
C	-5.940582	1.682965	0.658329
C	-5.649288	2.611295	-0.359793
C	-4.519503	2.442021	-1.147859
C	-3.681824	1.347778	-0.937523
O	-6.470139	3.670455	-0.561306
O	-7.070436	1.974449	1.358569
C	-7.444686	1.111843	2.424712
C	-4.557418	-2.363204	-1.067022

O	-4.596409	-3.248100	-2.165779
O	3.547617	-2.519624	1.642110
O	5.825998	-1.188517	2.040672
O	7.515728	0.664328	1.328270
O	6.049067	3.721565	-2.000086
H	3.896308	2.339270	-2.065771
H	7.797931	2.719079	-0.231173
H	6.923487	4.105647	-1.850380
H	7.142767	-0.129614	1.791689
H	2.985471	-0.532221	1.804259
H	-0.197329	-4.190428	-1.496743
H	2.172348	-3.445938	-1.307086
H	0.837454	0.238842	0.473123
H	-2.910685	-1.210902	-1.841159
H	-4.828164	-2.875639	-0.132734
H	-5.294467	-1.582070	-1.259797
H	-3.932727	-3.934994	-2.017855
H	-5.305936	-0.103639	1.674032
H	-4.304392	3.171610	-1.921349
H	-2.794588	1.231714	-1.551999
H	-8.361488	1.528387	2.839302
H	-7.635769	0.098185	2.056896
H	-6.669438	1.088583	3.197569
H	-7.188715	3.620578	0.088889
H	-3.368674	-1.314908	1.187162

H	3.742773	-1.725957	-0.900708
H	4.344120	-2.646635	2.186490
B	-1.386419	0.465380	1.890512
H	-2.487497	0.714145	2.302232
H	-0.753521	1.436096	1.556848
H	-0.772773	-0.348384	2.537443

61

Coordinates of Silybin A_O1-BF_3_DMF.xyz performed

C	-4.620521	-1.665665	-1.516247
C	-4.339736	-0.654936	-0.628200
C	-5.088656	-0.484650	0.557011
C	-6.198046	-1.344835	0.786508
C	-6.499683	-2.366493	-0.104497
C	-5.696946	-2.525661	-1.232929
O	-3.306041	0.224398	-0.949579
C	-2.992930	1.369551	-0.055466
C	-3.283072	0.996630	1.401297
C	-4.671862	0.436945	1.561378
C	-1.552124	1.745819	-0.235052
C	-0.550687	0.778905	-0.138693
C	0.787967	1.143148	-0.233629
C	1.133179	2.490095	-0.418703
C	0.134137	3.457087	-0.492610
C	-1.203378	3.087063	-0.407049

O	1.735082	0.164730	-0.151745
C	3.068409	0.629822	0.127285
C	3.372252	1.824559	-0.775735
O	2.435230	2.879163	-0.514225
C	4.014442	-0.519056	-0.073094
C	4.892913	-0.878385	0.958493
C	5.797537	-1.916851	0.771238
C	5.826847	-2.613804	-0.450861
C	4.950609	-2.262591	-1.468528
C	4.048415	-1.215339	-1.281483
O	6.707518	-3.632000	-0.627562
O	6.700117	-2.367499	1.684687
C	6.740402	-1.736830	2.959240
C	4.743483	2.432165	-0.542491
O	4.958452	3.560981	-1.369180
O	-3.101710	2.133960	2.196026
O	-5.333916	0.731945	2.573265
O	-6.946586	-1.193101	1.882319
O	-5.914952	-3.499155	-2.135091
H	-4.055311	-1.792956	-2.428210
H	-7.341215	-3.021782	0.092515
H	-6.670151	-4.045749	-1.876883
H	-6.581981	-0.425609	2.392375
H	-2.589008	0.194301	1.704077
H	0.421051	4.493932	-0.634164

H	-1.976088	3.845563	-0.480282
H	-0.787388	-0.271827	0.000343
H	3.269416	1.522195	-1.826968
H	4.850950	2.695323	0.519296
H	5.513833	1.701853	-0.796478
H	4.264443	4.204864	-1.173539
H	4.861141	-0.344018	1.901883
H	4.981258	-2.814557	-2.402318
H	3.361101	-0.954959	-2.080082
H	7.520128	-2.248119	3.521655
H	6.993030	-0.676060	2.861316
H	5.781922	-1.844942	3.477226
H	7.223569	-3.727193	0.188787
H	3.103134	0.967298	1.171687
H	-3.653186	2.187065	-0.359919
H	-3.827775	2.123859	2.844899
B	-3.135645	0.597305	-2.617935
H	-2.508867	-0.332400	-3.058939
H	-2.538869	1.636271	-2.596166
H	-4.280189	0.673580	-2.986864

61

Coordinates of Silybin A_O4-BF_3_DMF.xyz performed

C	-4.750792	-0.991836	-2.135337
C	-4.334317	-0.470443	-0.931826

C	-5.165221	-0.489083	0.248013
C	-6.533420	-0.910538	0.057775
C	-6.945274	-1.418855	-1.165486
C	-6.056395	-1.485824	-2.239539
O	-3.080900	0.028261	-0.907365
C	-2.823180	1.060007	0.066275
C	-3.131055	0.454172	1.422169
C	-4.542045	-0.115183	1.461492
C	-1.404548	1.519770	-0.092037
C	-0.356298	0.597499	-0.083108
C	0.961299	1.027671	-0.194989
C	1.239769	2.396446	-0.326753
C	0.196793	3.318035	-0.324469
C	-1.119644	2.882171	-0.210393
O	1.954937	0.092093	-0.183003
C	3.269992	0.609960	0.088118
C	3.494713	1.851018	-0.775343
O	2.519650	2.849887	-0.444903
C	4.266794	-0.482842	-0.169627
C	5.187760	-0.825119	0.830261
C	6.138891	-1.810150	0.591941
C	6.173137	-2.470419	-0.650282
C	5.254919	-2.136800	-1.636320
C	4.305597	-1.142805	-1.397994
O	7.099736	-3.436635	-0.877076

O	7.085637	-2.239044	1.470437
C	7.124001	-1.645274	2.762691
C	4.844077	2.510527	-0.558215
O	4.984191	3.677465	-1.347375
O	-2.942309	1.419099	2.420201
O	-4.955778	-0.234503	2.656977
O	-7.497883	-0.764509	0.962736
O	-6.417104	-1.979632	-3.432010
H	-4.090072	-0.983019	-2.993033
H	-7.978565	-1.730200	-1.272096
H	-7.337913	-2.277545	-3.419893
H	-7.188824	-0.704527	1.904276
H	-2.462472	-0.413038	1.574014
H	0.432017	4.372445	-0.426406
H	-1.926653	3.608338	-0.216588
H	-0.542213	-0.468314	0.008638
H	3.373801	1.584469	-1.834454
H	4.971963	2.739113	0.509242
H	5.637257	1.824851	-0.861468
H	4.266687	4.280128	-1.109715
H	5.152129	-0.319973	1.789532
H	5.290103	-2.660615	-2.586039
H	3.586223	-0.895806	-2.172147
H	7.943814	-2.129999	3.290567
H	7.316987	-0.569926	2.691932

H	6.185991	-1.820732	3.299407
H	7.639911	-3.527927	-0.076028
H	3.309929	0.912485	1.143075
H	-3.509166	1.896650	-0.119335
H	-3.384200	1.096935	3.220863
B	-5.968544	-1.190024	3.344743
H	-5.972870	-2.232729	2.727702
H	-5.587642	-1.295858	4.485264
H	-7.058216	-0.627553	3.347914

61

Coordinates of Silybin A_O9-BF_3_DMF.xyz performed

C	-5.108510	-0.962211	-1.953399
C	-4.611846	-0.469859	-0.762267
C	-5.337449	-0.608104	0.446820
C	-6.615300	-1.230984	0.411066
C	-7.124243	-1.728287	-0.780740
C	-6.359812	-1.595698	-1.945079
O	-3.401136	0.137167	-0.786052
C	-3.089696	1.032871	0.300331
C	-3.321169	0.299264	1.624608
C	-4.757696	-0.180983	1.677883
C	-1.674443	1.500783	0.113001
C	-0.656517	0.580194	-0.133059
C	0.655773	1.011422	-0.292488

C	0.949393	2.373017	-0.206592
C	-0.049963	3.295868	0.068235
C	-1.361500	2.857201	0.222130
O	1.635212	0.094484	-0.525452
C	2.899275	0.505507	0.015580
C	3.321539	1.829551	-0.609508
O	2.252479	2.830382	-0.430135
C	3.904134	-0.579865	-0.246611
C	4.652448	-1.100379	0.817783
C	5.591298	-2.097777	0.582724
C	5.781099	-2.593418	-0.720590
C	5.029839	-2.083979	-1.771031
C	4.096404	-1.075443	-1.535618
O	6.693067	-3.572916	-0.944847
O	6.383457	-2.688326	1.518076
C	6.266137	-2.258018	2.869200
C	4.589065	2.406845	0.017024
O	5.659259	2.145951	-0.872707
O	-3.035380	1.133881	2.712558
O	-5.345890	-0.200435	2.777902
O	-7.327662	-1.356962	1.536629
O	-6.796298	-2.057662	-3.133435
H	-4.555799	-0.848032	-2.877680
H	-8.097156	-2.208119	-0.794506
H	-7.666628	-2.469077	-3.042047

H	-6.795622	-0.949221	2.267844
H	-2.680624	-0.600129	1.637549
H	0.196795	4.348263	0.131446
H	-2.143432	3.582588	0.421949
H	-0.860952	-0.482459	-0.214213
H	3.458017	1.716402	-1.688312
H	4.447282	3.482266	0.165924
H	4.771352	1.947488	0.996952
H	6.487512	2.356039	-0.424876
H	4.492785	-0.726802	1.823527
H	5.183951	-2.481229	-2.768934
H	3.511458	-0.688412	-2.363790
H	6.985213	-2.848619	3.434648
H	6.509585	-1.194610	2.961432
H	5.256891	-2.443416	3.250753
H	7.110000	-3.794994	-0.097026
H	2.776523	0.646809	1.098106
H	-3.772226	1.891640	0.251196
H	-3.714864	0.943058	3.382794
B	2.407931	4.205181	-1.391717
H	3.536601	4.126360	-1.800559
H	1.558694	4.053623	-2.235587
H	2.218558	5.125070	-0.634713

61

Coordinates of Silybin A_O12-BF_3_DMF.xyz performed

C	4.639514	1.968315	-1.368483
C	4.384231	0.871625	-0.568188
C	5.338288	0.416314	0.375461
C	6.589150	1.086529	0.462769
C	6.855883	2.188850	-0.337286
C	5.874603	2.619997	-1.236279
O	3.186531	0.253113	-0.706616
C	3.090869	-1.110151	-0.244908
C	3.591639	-1.177101	1.200937
C	5.012904	-0.656558	1.256897
C	1.664083	-1.552630	-0.398486
C	0.626581	-0.743856	0.069160
C	-0.685227	-1.181088	-0.034185
C	-0.982691	-2.412105	-0.621470
C	0.051934	-3.220142	-1.081675
C	1.369773	-2.793333	-0.966881
O	-1.707781	-0.374254	0.477263
C	-3.115636	-0.797171	0.272677
C	-3.179619	-1.732981	-0.924712
O	-2.272824	-2.826042	-0.752852
C	-3.961954	0.425478	0.081119
C	-5.110059	0.593899	0.868058
C	-5.939057	1.687375	0.655025

C	-5.619451	2.632134	-0.339530
C	-4.477048	2.467178	-1.110340
C	-3.653247	1.360940	-0.905819
O	-6.427317	3.703097	-0.535786
O	-7.078931	1.975306	1.338260
C	-7.490092	1.087540	2.371687
C	-4.556613	-2.355067	-1.099726
O	-4.588443	-3.210102	-2.223554
O	3.523294	-2.487888	1.690170
O	5.809724	-1.166496	2.070254
O	7.512420	0.665495	1.334943
O	6.072054	3.682008	-2.042277
H	3.912940	2.309373	-2.095562
H	7.810956	2.696438	-0.255717
H	6.949025	4.063806	-1.898680
H	7.132925	-0.119567	1.808106
H	2.974059	-0.494377	1.810728
H	-0.196327	-4.172653	-1.537392
H	2.171488	-3.427308	-1.332294
H	0.829703	0.220315	0.519457
H	-2.901502	-1.192857	-1.840056
H	-4.831756	-2.893395	-0.182228
H	-5.293368	-1.569666	-1.275196
H	-3.941138	-3.913764	-2.081193
H	-5.342092	-0.126325	1.645022

H	-4.241339	3.207461	-1.867663
H	-2.758275	1.246373	-1.509218
H	-8.413097	1.503178	2.772794
H	-7.680686	0.086533	1.971539
H	-6.735638	1.035856	3.163205
H	-7.162575	3.644254	0.095277
H	-3.396313	-1.337426	1.181211
H	3.740951	-1.735932	-0.870525
H	4.321710	-2.612856	2.232543
B	-1.404841	0.426790	1.918368
H	-2.506073	0.652025	2.344615
H	-0.791193	1.414960	1.596217
H	-0.770552	-0.376230	2.559801

Table S4. Absolute energies of different compounds used for Fukui function calculation

Compound	Electronic State		EtOAc	DMF
Silybin A (**1a**)	Molecular	qk(N)	-1722.762612	-1722.76835
	Radical cation	qk(N-1)	-1722.515391	-1722.52913
	Radical Anion	qk(N+1)	-1722.865558	-1722.8816
Silybin B (**1b**)	Molecular	qk(N)	-1722.755298	-1722.762517
	Radical cation	qk(N-1)	-1722.513686	-1722.52755
	Radical Anion	qk(N+1)	-1722.857723	-1722.875788

Table S5. Absolute energies of different [BF_3-silybin A] complexes and BF_3 in different solvents

Compound		EtOAc	DMF
[BF_3-silybin A]	O1	-1749.525928	-1749.532411
	C4=O	-1749.535676	-1749.542037
	O9	-1749.526772	-1749.533848
	O12	-1749.533171	-1749.539618
BF_3		-26.75323207	-26.75323207

Typical used input line for optimization with Gaussian09 package

#p opt=(maxcycles=256) freq=noraman b3p86/6-31+G(d,p) scf=(xqc,maxcycles=500) pop=(minimal,espdipole) gfprint gfinput scrf=(iefpcm,solvent=ethylethanoate)
